# Differentially expressed transcripts and associated protein pathways in basilar artery smooth muscle cells of the high-salt intake–induced hypertensive rat

**DOI:** 10.7717/peerj.9849

**Published:** 2020-10-13

**Authors:** Junhao Huang, Lesha Zhang, Yang Fang, Wan Jiang, Juan Du, Jinhang Zhu, Min Hu, Bing Shen

**Affiliations:** 1 Guangzhou Sport University, Guangdong Provincial Key Laboratory of Sports and Health Promotion, Guangzhou, Guangdong, China; 2 School of Basic Medical Sciences, Anhui Medical University, Hefei, Anhui, China

**Keywords:** Basilar artery, Vascular smooth muscle cell, High-salt intake, Hypertension, High-throughput sequencing

## Abstract

The pathology of cerebrovascular disorders, such as hypertension, is associated with genetic changes and dysfunction of basilar artery smooth muscle cells (BASMCs). Long-term high-salt diets have been associated with the development of hypertension. However, the molecular mechanisms underlying salt-sensitive hypertension-induced BASMC modifications have not been well defined, especially at the level of variations in gene transcription. Here, we utilized high-throughput sequencing and subsequent signaling pathway analyses to find a two–fold change or greater upregulated expression of 203 transcripts and downregulated expression of 165 transcripts in BASMCs derived from rats fed a high-salt diet compared with those from control rats. These differentially expressed transcripts were enriched in pathways involved in cellular, morphological, and structural plasticity, autophagy, and endocrine regulation. These transcripts changes in the BASMCs derived from high-salt intake–induced hypertensive rats may provide critical information about multiple cellular processes and biological functions that occur during the development of cerebrovascular disorders and provide potential new targets to help control or block the development of hypertension.

## Introduction

Hypertension plays a substantial etiological role in cerebrovascular disorders, such as stroke, which have high incidence and mortality rates (Liu et al., 2007; Zhou et al., 2019). The pathology of most cerebrovascular disorders is associated with homeostatic changes and dysfunction of basilar artery smooth muscle cells (BASMCs). Vascular smooth muscle cells possess remarkable phenotypic plasticity that enables them to rapidly switch their phenotype in response to a variety of environmental stimuli (Starke et al., 2014). This phenotypic switching is a crucial component of vascular diseases, such as hypertension, and in aging (Lacolley et al., 2012). Homeostatic changes, especially an increase in the free intracellular calcium concentration ([Ca^2+^]_i_), not only determine the contractile state of vascular smooth muscle cells (VSMCs) but also impact the activities of multiple Ca^2+^-associated transcription factors and regulators, which in turn regulate the phenotype and function of VSMCs (Touyz et al., 2018).

It has already been shown that a long-term high-salt diet can lead to hypertension and to the development of cardiovascular disease (Grillo et al., 2019; Karppanen & Mervaala, 2006). High-salt intake is one of the high-risk factors contributing to cerebral ionic homeostatic impairment, especially for Ca^2+^, in VSMCs (Zhu et al., 2005). High-salt intake causes increased transient receptor potential polycystic 2 expression levels in VSMCs, thereby sensitizing the blood vessel response to contractors and inducing hypertension (Zhao et al., 2015). Arteries from rats fed a high-salt diet show reduced ability to properly modulate [Ca^2+^]_i_ levels, with increased activity of the Ca^2+^ sensitization pathway RhoA/ROCK (Crestani, Webb & Da Silva-Santos, 2017). Furthermore, a recent study has shown that in the isolated culture of human aortic smooth muscle cells, increasing NaCl concentration by as little as 4 mM causes substantial cell membrane damage, leading to chronic sodium (Na^+^) and Ca^2+^ overloads and hypertrophy in VSMCs (Bkaily et al., 2018). However, the molecular mechanisms underlying the salt-sensitive hypertension-induced VSMC modifications have not been well described. High-throughput sequencing technologies are efficient tools for examining genetic or genomic variation and for evaluating potential biomarkers in clinical research (Soon, Hariharan & Snyder, 2013). Transcripts changes in the high-salt intake–induced hypertension rat model, especially in BASMCs, may provide critical information about multiple cellular processes and biological functions that occur during the development of cerebrovascular disorders.

Therefore, in the present study, transcript expression levels in BASMCs derived from rats fed normal chow or a high-salt diet were identified by next-generation sequencing. Several bioinformatics tools were applied to analyze the data from those two groups. Their differentially expressed genes (DEGs) and associated protein pathways were identified to construct a protein-protein interaction (PPI) network and to determine potential key proteins. The results of this study highlight the significant molecular and signaling pathway alterations in BASMCs that are induced by high-salt intake, providing insights into potential new therapeutic strategies for cerebrovascular disorders associated with the dysfunction of VSMCs.

## Materials & Methods

### Animal treatment

All the animal experiment procedure was also permitted by the Animal Ethics Committee of Anhui medical university (Licience No. LLSC20150048). All procedures were conducted in accordance with our institutional guidelines and the National Institutes of Health Guide of the Care and Use of Laboratory Animals. The 6-week old male SD rats (180–200 g) were purchased from the animal center of Anhui medical university, then separately into two groups of high-salt group and control group. Rats were housed in 485 mm × 350 mm × 200 mm cages with three rats in each cage. The lights in the raising environment were maintained in a 12/12 h light/dark cycle. Temperature was controlled between 22−25 °C. The animals were fed under free food and water. The high-salt diet comprised rat chow supplied for control group rat and additional 4% (w/w) sodium chloride (NaCl). When the systolic blood pressure levels were determined by tail-cuff method and significantly increased above 30 mmHg in the rats eating the high-salt diet for 4 weeks, all these rats along with control rats fed a normal rat chow diet were sacrificed by continuously inhaling carbon dioxide, which was complied with the requirement of euthanizing animals criteria. The rats’ brains were isolated and put in the Kreb’s solution (NaCl 118 mM, KCl 4.7 mM, CaCl_2_ 2.5 mM, MgSO_4_ 1.2 mM, KH_2_PO_4_ 1.2 mM, NaHCO_3_ 25 mM, glucose 11.1 mM, pH 7.2–7.4, 4 °C). Brain basilar arteries were isolated, and then unnecessary connective tissues were eliminated by using anatomical microscope. By mechanically removed using wires repeatedly, the endothelial cells were denuded. All the tissues collective operation was previously described (Jiang et al., 2019). All the remain basilar artery smooth muscle cells of each single rat were collected, and then messenger RNAs extractions were performed using Poly A selection of Illumina TruSeq RNA sample preparation protocol to establish the RNA sample libraries.

### Blood pressure measurement by tail-cuff method

The systolic blood pressure of model rats was determined by a noninvasive method using tail-cuff plethysmography as previously described (Streeter, Hart & Badoer, 2012). Firstly, conscious rats were placed in a pre-warmed chamber for 15–20 min before blood pressure examination. Then, a pneumatic pulse transducer was settled on the rat’s tail. The signals from tails were conducted and analyzed automatically by a data recording and analysis system (BL-420E+, Chengdu Technology & Market Corp, China). Blood pressure of each rat was recorded as the mean of these measurements. Data was presented as means ± SEM, and statistical analysis was performed using two-way analysis of variance followed by Games-Howell *post hoc* tests by GraphPad Prim 6 (GraphPad Software Inc). Statistical significance was considered when *P < 0.05.

### High-throughput sequencing and data analysis

The RNA sample libraries were established and sequenced using the high-throughput Illumina HiSeq 2500 sequencing platform, with a sequencing length of 150 base pairs. We finally acquired three RNA sample libraries of the rats of control group and three RNA sample libraries of the rats of high-salt diet group, which were labeled as case group. All the quantity and quality of RNA sample were up to the standards of high-throughput sequencing. The RNA sequencing (RNA-Seq) Fastq raw data were pruned using Trimmomatic to remove adaptors and lower mass reads. The quality of clean data was assessed using FastQC software (Davis, Bardsley & Paterson, 2018). The quality-approved data were mapped to the reference genome of the rat (National Center for Biotechnology Information [NCBI] genome assembly, version Rnor_6.0) using HISAT2 (v2.0.13) (Pertea et al., 2016) and annotated with the annotation file (.gtf) NCBI Rnor_6.0. The transcript expression levels were then calculated using fragments per kilobase of transcript per million fragments mapped. The RNA-Seq quantification software Kallisto (Bray et al., 2016) was used to obtain a count of known messenger RNAs (mRNAs) to compare the differences in transcript expression. Using the edgeR package software (http://bioconductor.org/packages/2.4/bioc/html/edgeR.html) (Robinson, McCarthy & Smyth, 2010), the differences in the number of reads among samples were analyzed. The significance levels between the genetic transcripts of the two experimental groups were calculated using a negative binomial model and were assessed using an exact test analogous to Fisher’s exact test. Gene Ontology (GO) and Kyoto Encyclopedia of Genes and Genomes (KEGG) analyses were performed on differentially expressed transcriptomes with a 2 fold change cutoff.

### GO and KEGG pathway enrichment analyses

The functional pathway enrichment of the proteins encoded by DEGs was analyzed and annotated using the Metascape database. The Metascape online tool was applied to the screened transcripts to develop the GO annotations. The KEGG pathway analysis was also performed using the same database. RNA-Seq data were used to determine the DEGs that were significantly upregulated or downregulated, with a two-sided *P* value <0.05 considered statistically significant. In addition, the GO and KEGG pathway enrichment analyses were also statistically tested with a two-sided *P* value <0.05 considered statistically significant.

### PPI network integration and module analyses

PPI analysis can provide potential protein functions that are useful for determining the general organization principles of protein functional networks. The online search tool STRING (http://string.embl.de/) (Szklarczyk et al., 2019) was used to discover related genes/mRNAs/proteins. We constructed respective PPI networks of high-expression transcripts and of low-expression transcripts to predict interactions using Cytoscape (http://www.cytoscape.org/) (Su et al., 2014) with a two-sided *P* value <0.05 considered statistically significant. Most previously acquired biological networks have been found to be subject to scale-free attribution. We selected modules using the Cytoscape (version 3.6.0) plugin MCODE (molecular complex detection) with MCODE scores ≥3 and with three or more nodes in the PPI network. The connectivity of the nodes in the PPI network was analyzed using topology analysis to obtain a higher degree of important nodes (i.e., central proteins). Detailed information from the top-10 hub gene transcripts is shown. The functional enrichment analysis of the individual modules was performed using Metascape, with a threshold for determining statistical significance set at *P* <0.05.

### qPCR (Quantitative real-time PCR)

qPCR experiments were conducted to test mRNA expression change of the most differential expression 10 hub gene transcripts in BASMCs of high-salt induced hypertension rats compared to rats in control group. After extracting RNA, we added total RNA into a kit of HiScript III RT SuperMix for qPCR (Vazyme, China), then cDNA was synthesized. Total RNA (4 µL), 4 × gDNA wiper Mix (4 µL) and RNase-free ddH_2_O (8 µL) were mixed together, then the mixture was heated in a water bath (42 °C, 2 min). With HiScript III qRT SuperMix, qPCR reaction finished in the Roche LightCycler 480 II PCR equipment (Roche, Switzerland) with thermocycler steps of 5 min at 95 °C, 40 cycles of 15 s at 95 °C and 30 s at 60 °C. All the primer sequences used were listed in Table 1. *Gapdh* was used as a control. To analyze relative gene expression, 2^−ΔΔCT^ of each item were calculated and compared.

**Table 1:** The primer sequences used in qPCR. The forward and reverse primer sequences which were used in qPCR experiments to test mRNA expression change of the most differential expression 10 hub gene transcripts in BASMCs of high-salt induced hypertension rats compared to rats in control group and control gene GAPDH as well.

**Gene**	**Forward primer**	**Reverse primer**
*Akap9*	5′-GAAAAGCAAACGCAGACGGT-3′	5′-CGCTCGCTCTGTAAGTCGAT-3′
*Clasp1*	5′-GAACACACATTGGTGGTGCC-3′	5′-CAAGTCTGTGCACCAAGCAC-3′
*Egf*	5′-TGTGTCGGGAAGGATTCGTG-3′	5′-CACAGTGATGTCGTGCCTCT-3′
*Fyn*	5′-TAGTCGTGGCAAAAGGTCAGT-3′	5′-CCTCCTCTAGTACGCAGGGT-3′
*Gapdh*	5′-TGTGAACGGATTTGGCCGTA-3′	5′-GATGGTGATGGGTTTCCCGT-3′
*Gja1*	5′-GCTCCACTCTCGCCTATGTC-3′	5′-ACCCTAGGTGCATGTTCTGC-3′
*Igf1*	5′-TGTACTGTGCTCCGCTGAAG-3′	5′-CGGTGACGTGGCATTTTCTG-3′
*Mdc1*	5′-AGGATCTGTGCCTTTCAGCC-3′	5′-TCATGGGTGTCAATGGGCTC-3′
*Mmp9*	5′-GATCCCCAGAGCGTTACTCG-3′	5′-GTTGTGGAAACTCACACGCC-3′
*Pafah1b1*	5′-GAGTGACTCTTGCTGGGGAAA-3′	5′-TGGCCCATTAGAGGGGAGAA-3′
*Tp53*	5′-CCAGCTACCCGAAGACCAAG-3′	5′-GAGGGGGCCGAGTACTATCT-3′

## Results

### High-salt intake–induced changes of systolic blood pressure

**Figure 1:**
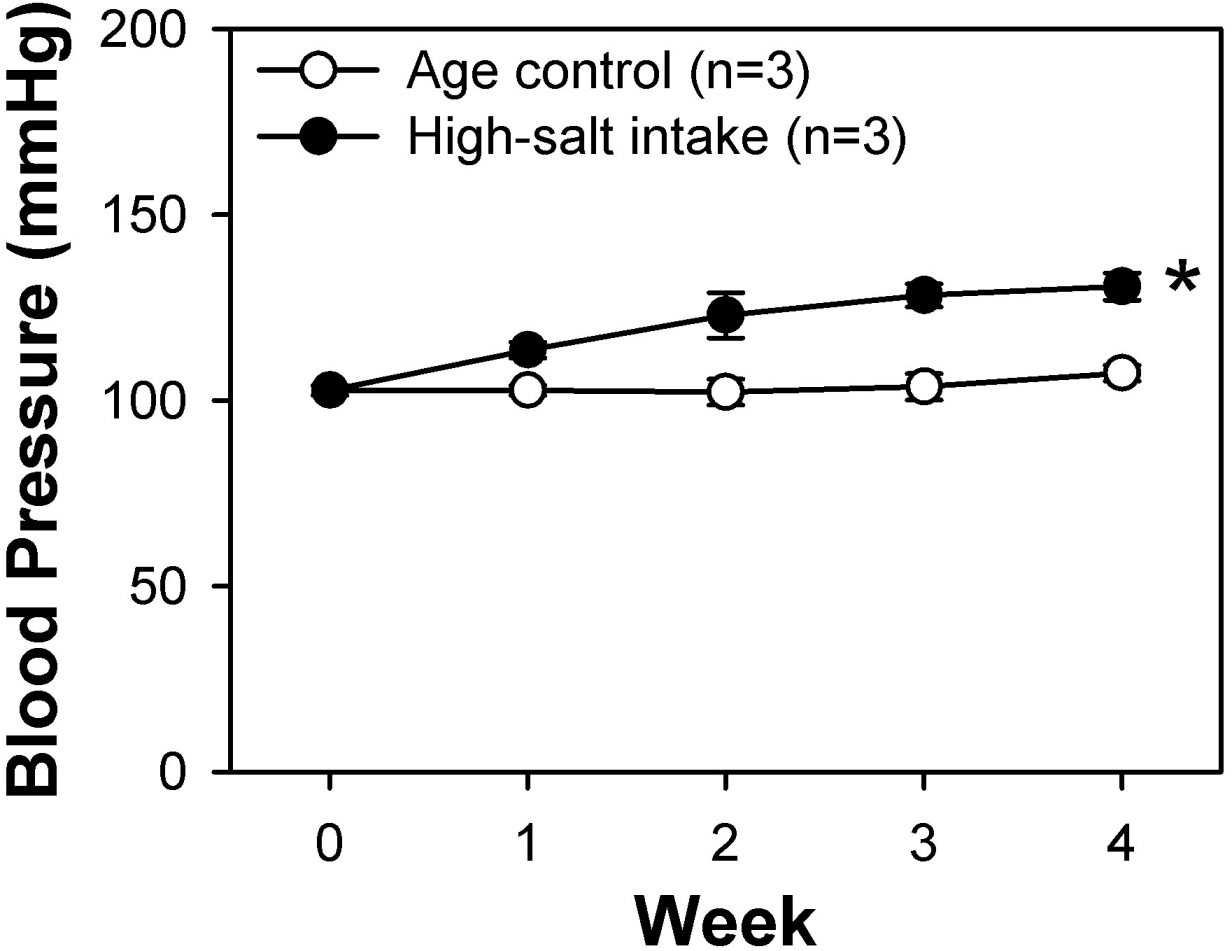
Systolic blood pressure weekly measured by tail-cuff method of control and high-salt intake rats. Values are showed with means ± SEM; *n* = 3 for each group. *P < 0.05 vs. control (two-way analysis of variance followed by Games-Howell post hoc tests).

The high-salt diet comprised rat chow containing 4% (w/w) sodium chloride (NaCl). Systolic blood pressure in the high-salt diet and control rats was monitored weekly by tail-cuff method. At the fourth week, we found that the systolic blood pressure of the rats intaking the high-salt diet was significantly higher than that of the rats of control group (Fig. 1). These rats along with control rats fed a normal rat chow diet were sacrificed and their BASMCs were isolated.

### Identification of differentially expressed transcripts in BASMCs

**Figure 2:**
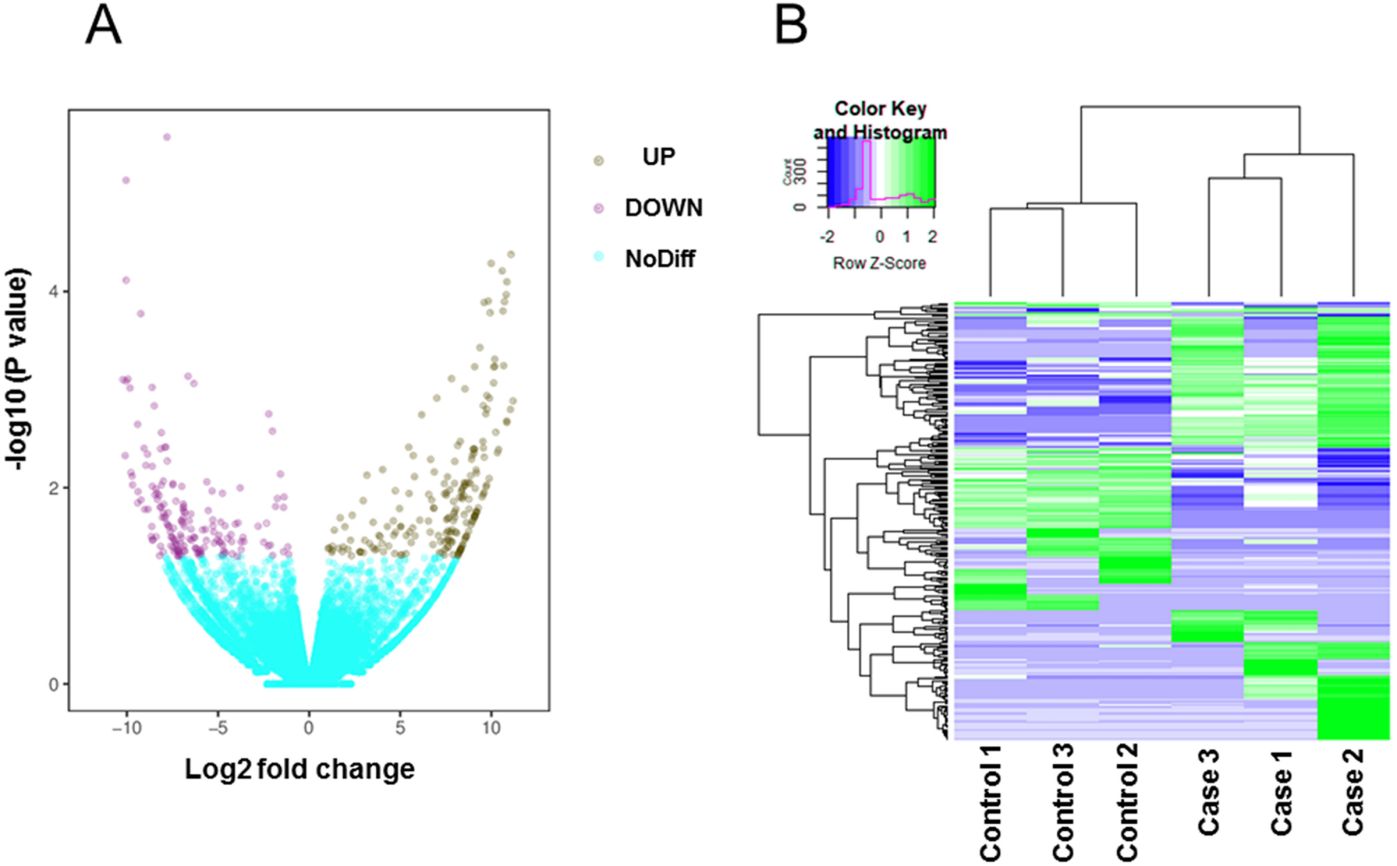
Transcripts differentially expressed by rats fed a normal diet (Control) and those fed a high-salt diet (Case). (A) Volcano map. Data are displayed on the basis of ≥2-fold change and a corrected *P* < 0.05. Purple dots represent upregulated transcripts (UP); teal dots, downregulated transcripts (DOWN); yellow dots, no significant difference (NoDiff). (B) Heat map showing the most significantly (*P* < 0.05) upregulated and downregulated transcripts based on fold changes.

Total mRNA from both rat groups was analyzed via high-throughput sequencing. A principal components analysis (PCA) indicated that the heterogeneity of RNAseq data between control and high-salt diet groups is obvious (Fig. S1). After using Kallisto software to accurately and quickly analyze the abundance of all the expressed transcripts, we used edgeR to analyze the differences among the transcripts. All the raw data and differently expressed gene transcripts information can be acquired and are accessible via GEO accession numbers series GSE147945. As shown on the volcano map (Fig. 2A), on the basis of an absolute fold change in expression levels of two or more and a corrected *P* < 0.05, we found that among the transcripts screened between the two groups of rats, the expression of 203 transcripts were significantly upregulated and the expression of 165 transcripts were significantly downregulated in the rats fed the high-salt diet. Additional details of all the 368 significantly changed transcripts were referred to Table S1. A heat map showing the cluster analysis of the different expression of the transcripts between two sets of samples is shown in Fig. 2B.

### GO functional enrichment analysis

**Figure 3:**
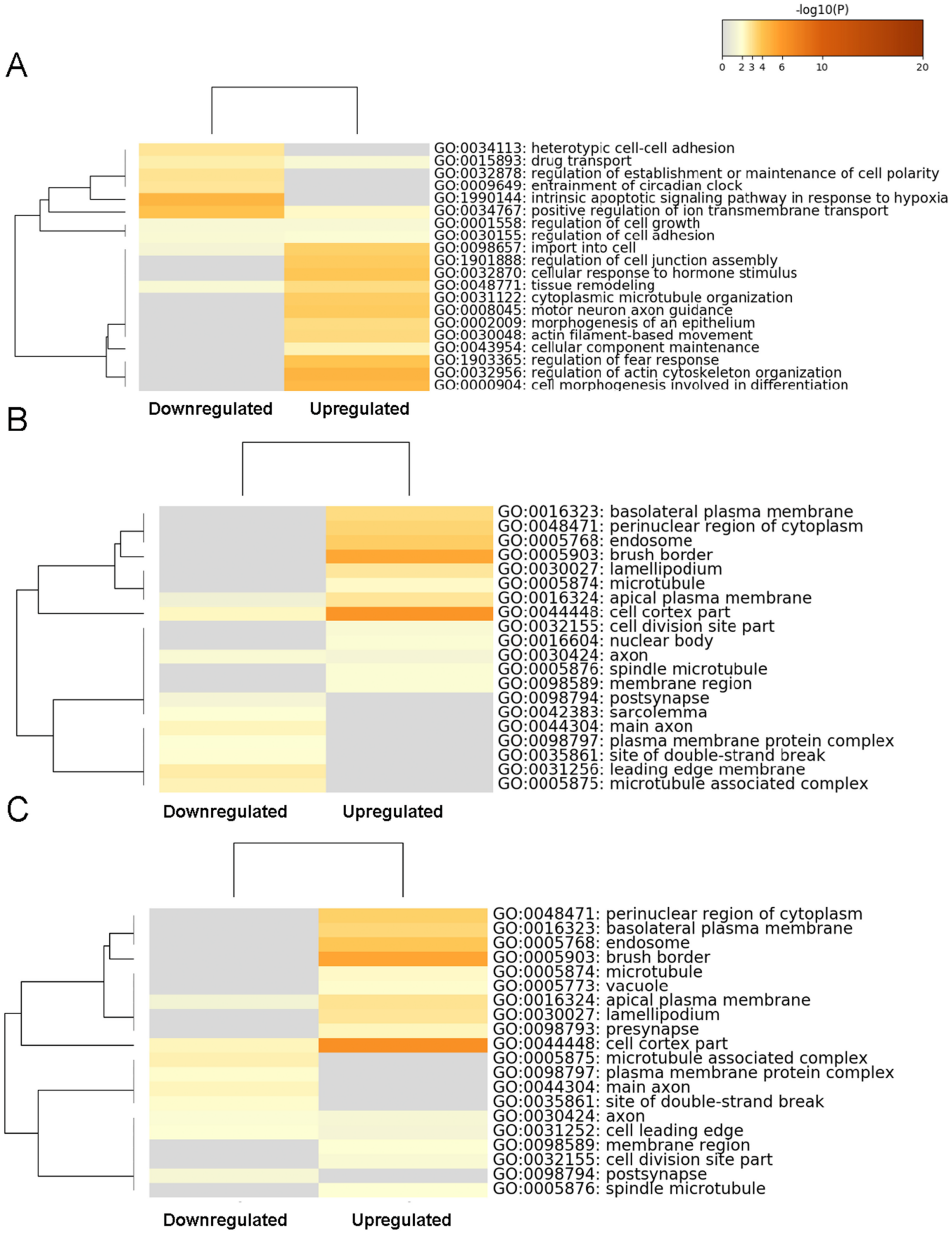
Heatmap of the top 60 enriched terms as analyzed by Metascape. Heat maps of the top 20 enriched terms in (A) biological processes, (B) cell composition, and (C) molecular function are shown and are colored according to their respective *p* values.

The top 60 significantly enriched terms were determined by conducting a GO functional enrichment analysis. The top 20 enriched biological processes (Fig. 3A), cell composition (Fig. 3B), and molecular function (Fig. 3C) terms are shown as heat maps. The biological processes GO category included enrichment for actin filament-based movement, cytoplasmic microtubule organization, tissue remodeling, import into cell, motor neuron axon guidance, regulation of actin cytoskeleton organization, cell morphogenesis involved in differentiation, regulation of fear response, and cellular response to hormone stimulus, indicating that those genes mostly control the cellular conformational plasticity in response to environmental changes. In the cell composition GO category, transcripts expression increased moderately and showed enrichment (but not in the top 20 items) for ion channel binding, protein-containing complex scaffold activity, and sulfur compound binding, whereas the more substantially upregulated gene transcripts in molecular function GO category showed enrichment in the cell cortex part and brush border, relating to the particular location of the changed gene transcripts. For the significantly lower-expression transcripts, our heat map findings indicated enrichments in the biological processes GO category for the intrinsic apoptotic signaling pathway. These findings suggested that the upregulated genes in the BASMCs may play roles associated with a high-salt diet.

### KEGG pathway analysis

**Figure 4:**
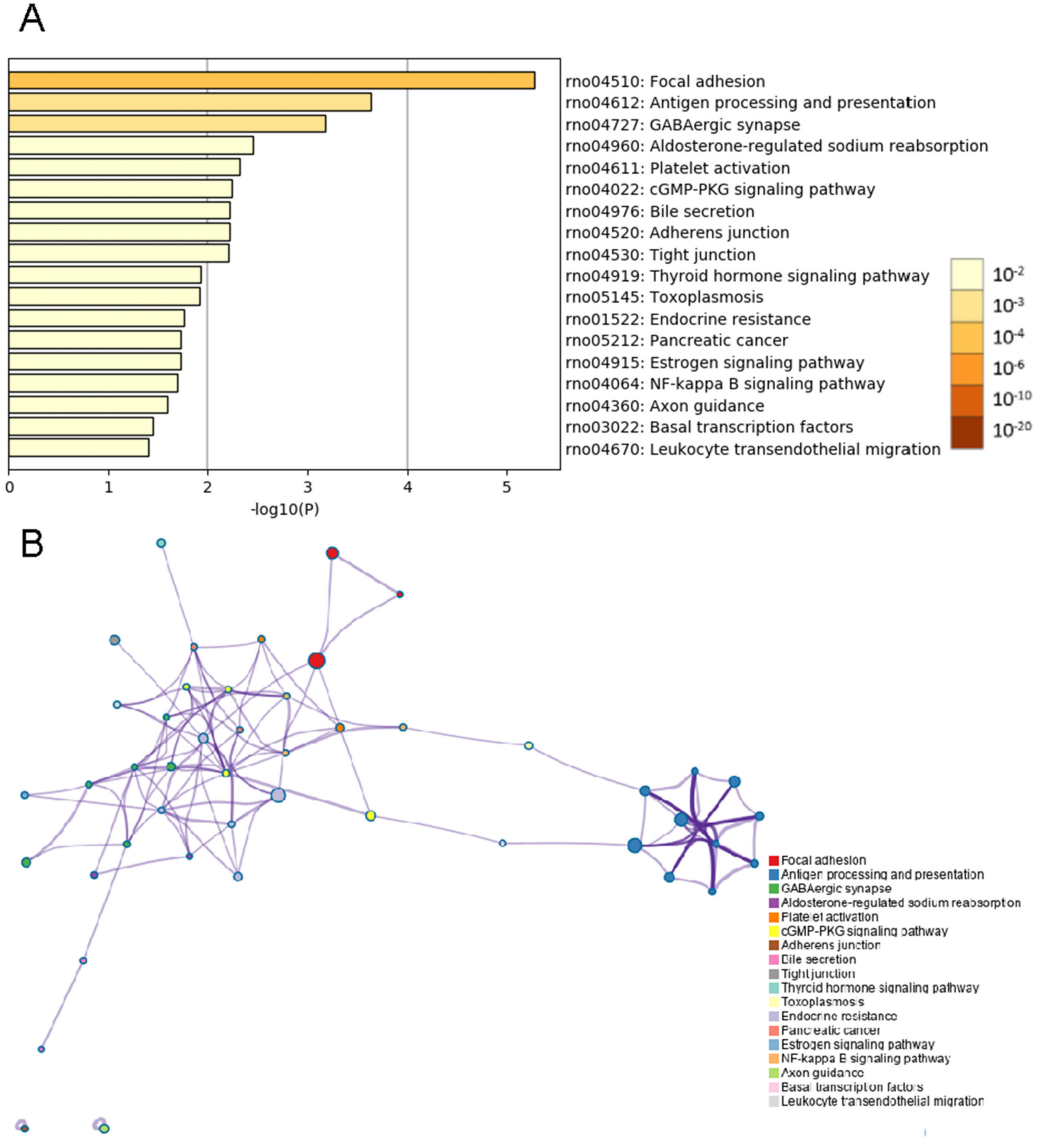
KEGG enrichment analysis by Metascape. (A) Heatmap of KEGG enriched terms across common genes differentially expressed between the high-salt diet experimental group and the control group. Colors represent *p* values. (B) Network of enriched terms is colored by cluster identification, and nodes sharing the same cluster are typically close to one another.

Analyses of whole mRNA sequences in the BASMCs derived from rats fed a high-salt diet and from control rats fed normal chow were conducted using the Metascape online analysis tool. The top 18 KEGG pathway enrichment analysis results indicated that the differential expression of transcripts were enriched in focal adhesion, antigen processing and presentation, GABAergic synapse, aldosterone-regulated sodium reabsorption, platelet activation, cGMP-PKG signaling pathway, bile secretion, adherens junction, tight junction, thyroid hormone signaling pathway, toxoplasmosis, endocrine resistance, pancreatic cancer, estrogen signaling pathway, NF-kappa B signaling pathway, axon guidance, basal transcription factors and leukocyte transendothelial migration (Fig. 4).

### PPI network construction, module analysis

**Figure 5:**
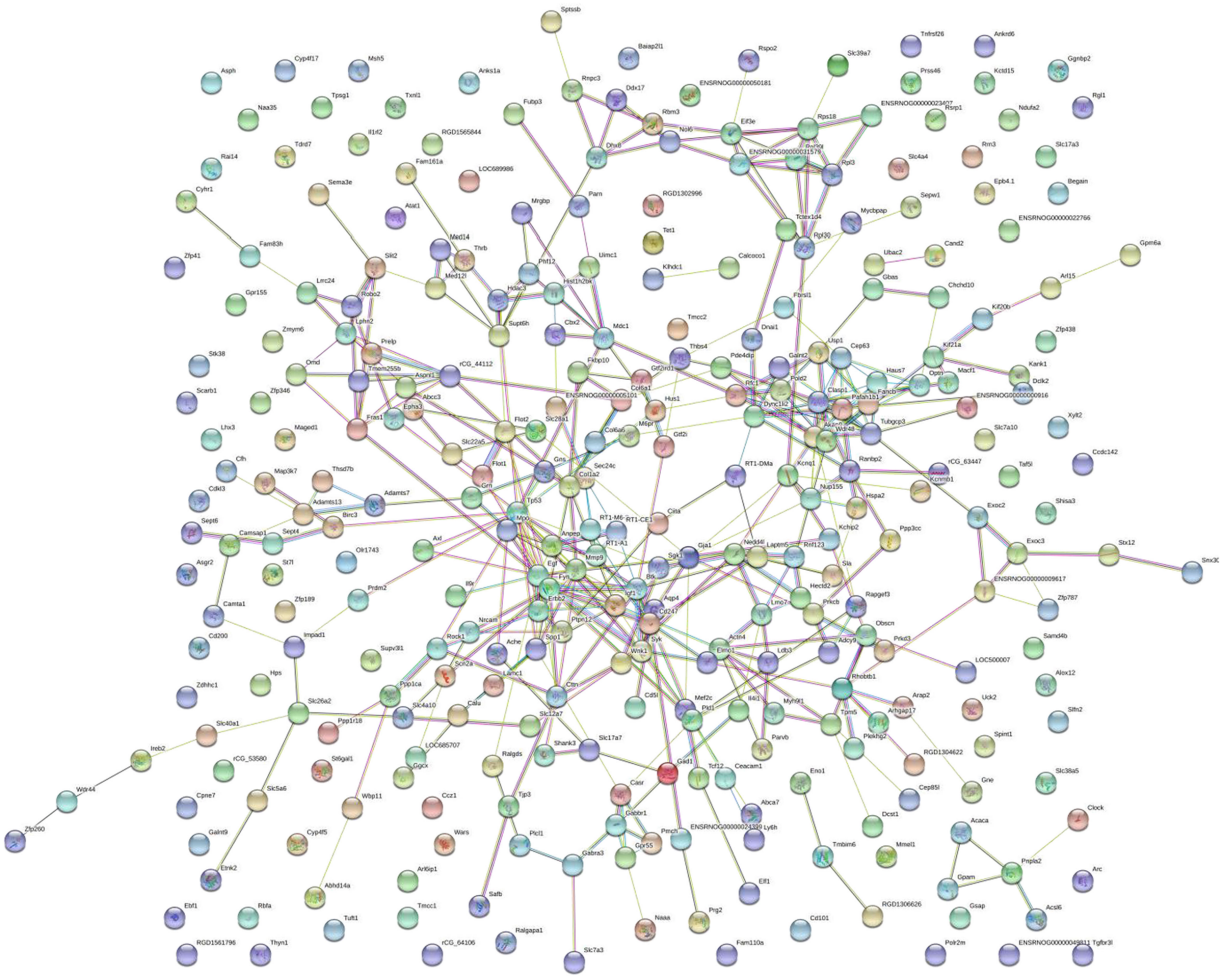
Protein–protein interaction network. Circles represent genes, lines represent protein interactions between genes, and line colors represent evidence of interactions between proteins.

**Figure 6:**
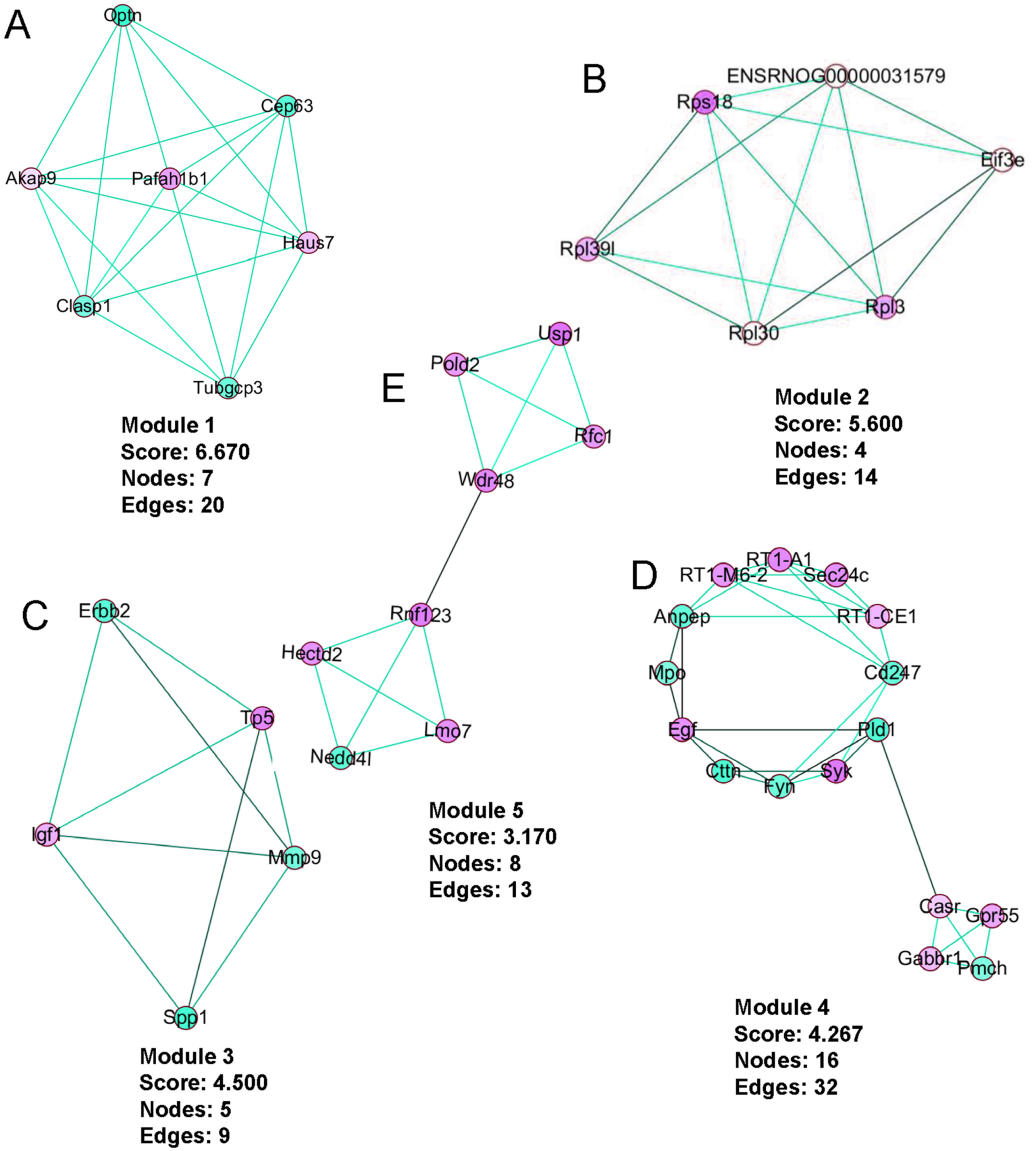
The five protein interaction modules analyzed by MCODE in Cytoscape. Upregulated genes are presented in red, downregulated genes are in green, and line colors represent evidence of interactions between proteins.

The PPI network was created using the STRING website (Fig. 5). We then used the MCODE plugin in Cytoscape to analyze the functional modules, and five of these modules are displayed in Fig. 6. Among the five functional modules, Module 1 was related to microtubules (Fig. 6A), and Module 2 was associated with protein translation and ribosomes (Fig. 6B). Module 3 was involved in tissue remodeling and the regulation of the cellular growth (Fig. 6C). The proteins presented in Module 4 predominantly contributed to regulation of the phospholipase D pathway and the ERK1/2 transduction cascade signaling pathway (Fig. 6D). The proteins of Module 5 participated in protein ubiquitination and hydrolysis (Fig. 6E). Through this PPI network construction and module analysis, we provided many key and close associated proteins, which may be importantly involved in the occurrence and development of high-salt induced hypertension.

### Hub gene transcripts selection and qPCR verification

**Figure 7:**
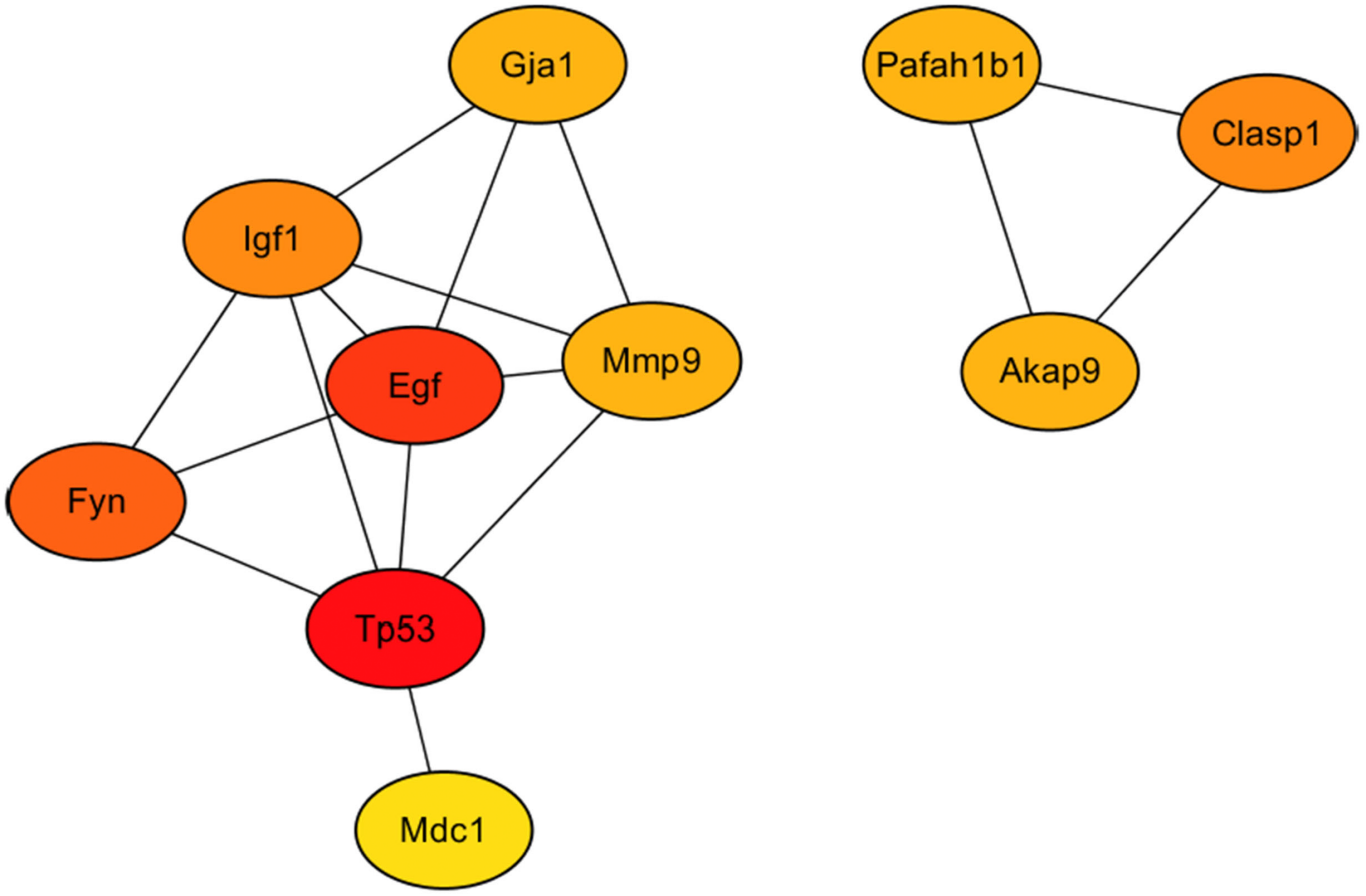
The 10 hub genes with the most substantial interactions according to the results of the CytoHubba plugin in Cytoscape. Protein-protein interaction network of the 10 hub genes. Circles of differently expressed genes are colored according to the protein-protein interaction calculation scores, with higher scores in red, middle-range scores in orange, and lower scores in yellow.

As showed in Fig. 7, the 10 hub gene transcripts with the most substantial interactions, according to the results of the CytoHubba plugin in Cytoscape (Chin et al., 2014), were *Akap9, Clasp1*, *Egf*, *Fyn*, *Gja1*, *Igf1*, *Mdc1*, *Mmp9*, *Pafah1b1* and *Tp53*, of which four gene transcripts were highly expressed and six genes transcripts showed low expression. Additionally, in Table 2, details of the 10 hub genes, including the logFC value which indicated the log of the fold change, were displayed. Therefore, through hub gene analysis, we provided 10 crucial genes possibly involving in the occurrence and development of high-salt induced hypertension, and their associated relationship.

**Table 2:** Details information of the 10 hub genes, including changes, the logFC value which indicated the log of the fold change and *P* value. Significance was statistically performed by comparing with regular diet control group.

**Name**	**Change**	**logFC**	***P*Value**
*Akap9*	Down-regulated	−2.339949944	0.023025188
*Clasp1*	Down-regulated	−7.931111523	0.003872411
*Egf*	Down-regulated	−7.931111523	0.003872411
*Fyn*	Up-regulated	7.822006234	0.028484544
*Gja1*	Up-regulated	1.039164861	0.041530831
*Igf1*	Down-regulated	−5.613259989	0.008720045
*Mdc1*	Up-regulated	8.381516052	0.010521468
*Mmp9*	Up-regulated	7.617054295	0.034324849
*Pafah1b1*	Down-regulated	−6.811791473	0.044484847
*Tp53*	Down-regulated	−8.596552153	0.033844722

**Notes.**

Significance was statistically performed by comparing with regular diet control group. LogFC indicates the log of the fold change.

**Figure 8:**
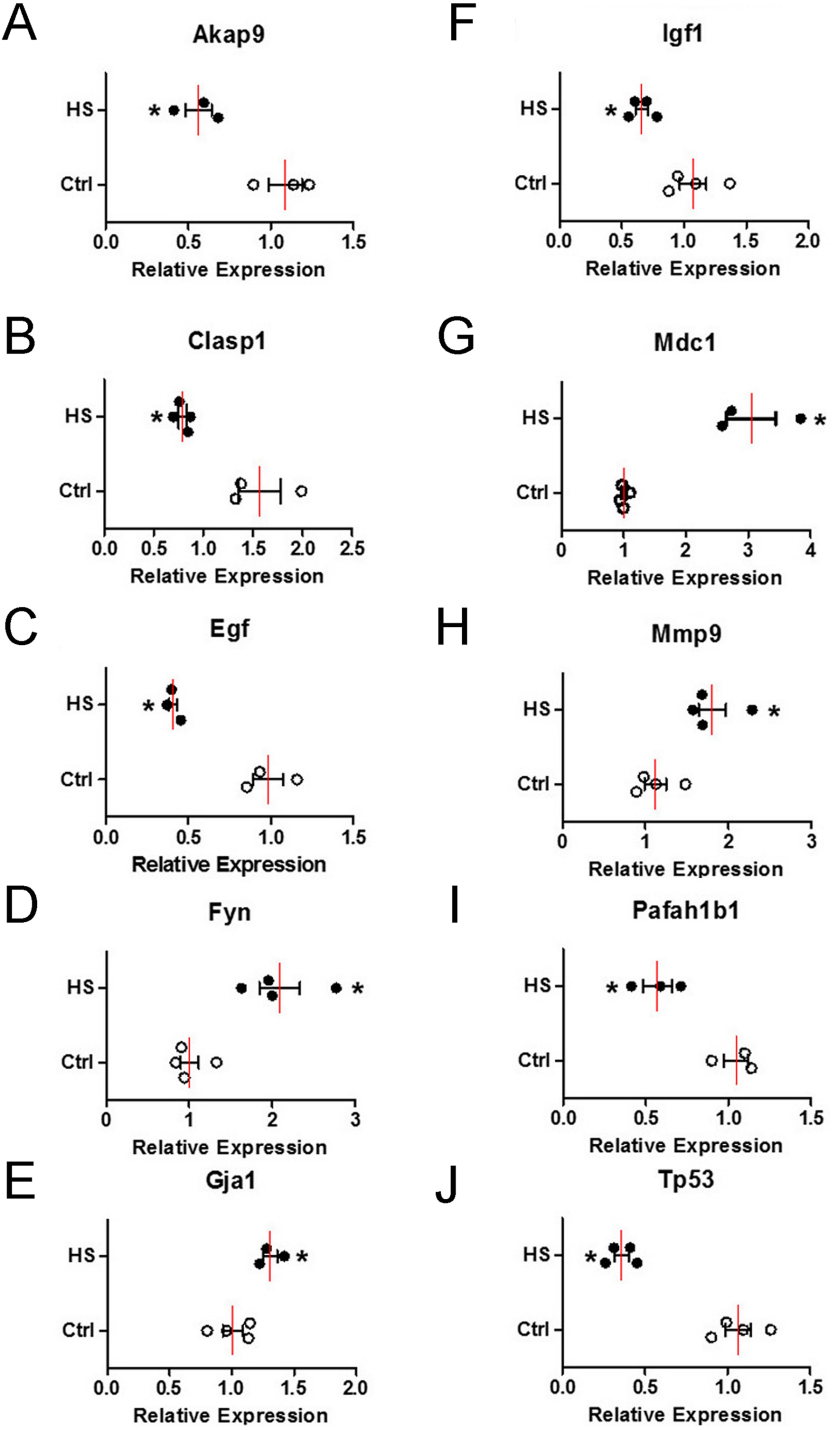
The mRNA relative expression levels of 10 hub genes. Four upregulated and six downregulated genes in BVSMCs of control (Ctrl) and high-salt diet induced hypertension (HS) rats. Values are shown as the mean ± SEM (*n* = 4 in each group). *P < 0.05 for HS vs. Ctrl group.

To verify the change of those 10 hub genes transcripts, we used qPCR (Quantitative Real-time PCR) to analysis the mRNA of each gene transcripts. According to the results in Fig. 8, the expression levels of four upregulated genes transcripts (*Fyn, Gja1, Mdc1 and Mmp9*) were significantly increased (Figs. 8D, 8E , 8G and 8H), while six downregulated genes transcripts (*Akap9, Clasp1, Egf, Igf1, Pafah1b1 and Tp53*) were significantly decreased (Figs. 8A–8C, 8F, 8I and 8J) in high-salt induced hypertension group compared to control group. The qPCR results indicated that the top differential change hub genes transcripts selection from RNA-Seq can be trusted.

## Discussion

Hypertension is prevalent worldwide and causes numerous related cerebrovascular complications (Iadecola & Davisson, 2008). The pathogenesis of hypertension is complex, with multifactorial genetic inheritance combined with environmental factors (Padmanabhan & Joe, 2017). Among all the environmental factors, high-salt intake as a dietary habit has already been shown to be involved in the development of hypertension. Short- or long-term high-salt treatment induces hypertrophy of human VSMCs, which then reduces the lumen of arteries, leading to an increase in blood pressure (Bkaily et al., 2018). Moreover, alterations in stiffness and adhesive properties of VSMCs contribute to the increased aortic stiffness occurring with hypertension superimposed on aging (Sehgel et al., 2015). VSMC phenotypic plasticity has more recently become known as a mechanism contributing to high-salt intake–induced hypertension (Zhao et al., 2015; Nickenig et al., 1998). As early as year of 1999, researchers have found that the stroke-prone spontaneously hypertensive rat (SHRSP) model with high incidence of cerebrovascular accidents showing structural modification included decreased lumen size, increased wall thickness without net growth and so on. These alterations in SHRSP rat basilar structure could be further exaggerated by increasing salt-loaded diet (Arribas et al., 1999). Therefore, it is necessary to discover the molecular variations of VSMCs in response to NaCl treatment.

Over the last decade, genomics has played an important role in understanding both the development of the pathogenesis and the therapies of disorders. The use of gene chips and next-generation sequencing has facilitated the discovery of causative genetic variants. These technologies have been widely used to study potential molecular mechanisms and to evaluate therapeutic targets and biomarkers (Russo et al., 2018). Despite numerous basic studies reporting on hypertension in recent years, the specific molecular and genetic mechanisms associated with VSMCs have not been elucidated because most of the previous studies have focused on genes associated with organ dysfunction (Xu et al., 2018). In the present study, we compared the changes in transcript expression levels in BASMCs between rats that modeled high-salt–induced hypertension and control rats. We found significantly upregulated and downregulated gene transcripts in the rats modeling hypertension. We analyzed these DEGs and constructed PPI networks to clarify associated protein pathways (Fig. 6). Finally, we determined 10 hug geneswith the highest degree of connectivity (Fig. 7). To our knowledge, this is the first study to report potential important genes and signaling pathways in VSMCs associated with a high-salt-induced hypertension disease.

According to the results of the top 60 significantly enriched terms determined by GO functional enrichment analysis, the upregulated gene transcripts enriching in actin filament-based movement, cytoplasmic microtubule organization, tissue remodeling and so on, which are primarily related to cellular morphological and structural conformation. These findings were consistent with the KEGG pathway analysis results showing enrichment in the pathways of focal adhesion, adherens junctions, and tight junctions. By contrast, the gene transcripts with significantly lower expression enriched in the GO category of biological processes were involved in the intrinsic apoptotic signaling pathway. Convincing evidence has shown that autophagy in VSMCs occurs in hypertension (Wolinsky et al., 1973), and treatment with an autophagy inhibitor restores the loss of p62 in VSMCs in a pulmonary hypertension model (Long et al., 2013). Results from our KEGG analysis indicated that endocrine regulation was changed in response to the increased Na^+^ intake, such as aldosterone-regulated Na^+^ reabsorption, the thyroid hormone signaling pathway, the estrogen signaling pathway, and endocrine resistance. Another group previously reported that significant upregulation occurs in several genes of *Fejervarya cancrivora*—frog. The gene expression changes in this frog are useful for enhancing their ability to thrive in a high salt environment. The upregulated genes are associated with aldosterone-regulated Na^+^ reabsorption pathways, which are related to osmotic regulation, enhancing their tolerance for a high salt environment (Shao et al., 2015). Another changed signaling pathway in our study in the high-salt–induced hypertensive male rats is the estrogen signaling pathway (Fig. 4). It is well known that cardiovascular dysfunction is widely found in hypertension disease. Estrogen, as known as one of the hormones that involved in energy homeostasis, involves in the development of hypertension by regulating the immune and vascular systems (Taylor & Sullivan, 2016). However, for the difference of male and female mammals in sex hormone regulation in pressure and cerebral blood flow (Barnes, 2017), it is necessary to research genetic transcripts changes in female animals and compare the gender difference.

The functional protein interaction indicated by the analysis of the PPI network construction, module analysis, and transcript-corresponding hub gene candidate showed that two PPI modules played a crucial role in high-salt–induced BASMC variations. The 10 hub genes candidate were *Akap9, Clasp1*, *Egf*, *Fyn, Gja1*, *Igf1*, *Mdc1, Mmp9*, *Pafah1b1* and *Tp53*, of which four gene transcripts were highly expressed and six showed low expression. The module containing *Akap9, Clasp1* and *Pafah1b1* was associated with cellular conformation plasticity. Akap9 is A-kinase anchor protein 9 (and is also known as centrosome- and Golgi-localized protein kinase N-associated protein or AKAP350 or AKAP450); it participates in the regulation of microtubule dynamics. Disrupting the AKAP9 interaction with short myomegalin-like EB1-binding protein (SMYLE) or with EB1 can inhibit microtubule nucleation and their stabilization at the leading edge of migrating cells (Bouguenina et al., 2017). Researchers have found that *Akap9* mutations markedly enhance the phosphorylated Tau: Tau ratio in lymphoblastoid cell lines treated with the phosphodiesterase-4 inhibitor rolipram, revealing the impact of AKAP9 mutations on Tau protein, considered a central mechanism in the pathogenesis of Alzheimer disease (Ikezu et al., 2018). *Clasp1* encodes a microtubule-stabilizing protein that can suppress the transition from microtubule growth to shortening (Aher et al., 2018) and is involved in the regulation of microtubule dynamics during neurite/axon outgrowth (Sayas et al., 2019). *Pafah1b1* is named for platelet-activating factor acetyl hydrolase 1B1, which is significantly expressed in the vascular system. It has been reported that a 17p deletion distal to *Pafah1b1* has a distinctive phenotype: mild intellectual disability, moderate to severe growth restriction, white matter abnormalities, and developmental defects (Schiff et al., 2010). In vascular samples from patients with chronic thromboembolic pulmonary hypertension, *Pafah1b1* expression is upregulated (Josipovic et al., 2016). However, in our sequencing results, *Pafah1b1* was downregulated in the rats fed the high-salt diet.

The second module, which comprised *Egf, Fyn, Gja1, Igf1, Mdc1, Mmp9 and Tp53*, was related to kinase signaling and cellular proliferation. Goetsch et al. utilized transcriptional profiling strategies and reported coordinated expression of *Egf, Igf1* and *Mmp9* during muscle regeneration (Goetsch et al., 2003). Proto-oncogene tyrosine-protein kinase Fyn is a member of the Src family of kinases playing a critical role in T-cell and neuronal signaling. Mechanical cyclic stretch contributing to hypertension can transiently induce translocation of caveolin from caveolae to noncaveolar membrane sites in BASMCs. When caveolin translocates to noncaveolar sites, it mediates ERK activation through combination with beta1-integrins/Fyn/Shc (Kawabe et al., 2004). Fyn is also a required intermediate, involved interaction between Ca^2+^/calmodulin-dependent protein kinase II *δ*2 in VSM cells and regulate its motility (Ginnan et al., 2013). Gja1 is gap junction alpha-1 protein (also known as connexin 43); it enables gap junction intercellular communication between cells to regulate cell death, blood pressure, and cardiac development (Nielsen et al., 2012). Mislocalization or dysfunction of Gja1 because of hypertension-induced myocardial remodeling might cause lethal arrhythmias and heart failure (Boengler & Schulz, 2017; Egan Benova et al., 2016; Basheer et al., 2015). Recent findings indicate that downregulated expression of myocardial Gja1 may be involved in this process (Egan Benova et al., 2016). However, as opposed to this finding, the present study reported that *Gja1* was upregulated after high-salt intake. The conflicting results may attribute to the distinct types of muscular cells, such as myocardial cells and VSMCs. Similar results of enhanced expression of Cx43 were also found in VSMCs of spontaneously hypertensive rats (Wang et al., 2016). Gja1, i.e., Cx43 hemichannels, also associates with Ca^2+^ transmission within vessel smooth muscular cells contributing to the Ca^2+^ dynamics and contractility of smooth muscular cells, by facilitating Ca^2+^ entry and participating control of vessel diameter and blood flow (Bol et al., 2017). High-salt intake-induced hypertension increases TRPP2 expression in VSMCs (Zhao et al., 2015), which may in turn change Ca^2+^ dynamics as well as the expression of Gja1. Mediator of DNA damage checkpoint protein 1 (Mdc1) is a regulator of the intra-S phase and the G2/M cell cycle checkpoints, recruiting repair proteins to the site of the damaged DNA. A recent study has shown that Mdc1 is involved in high salt-induced activation of the osmoprotective transcription factor osmotic response element-binding protein (also known as tonicity enhancer-binding protein) (Kunin et al., 2010). *Tp53* is a widely studied tumor suppressor gene. It has been shown that *Tp53* is upregulated during in vitro differentiation of mouse embryonic stem cells into smooth muscle cells, indicating a regulatory role in smooth muscle cell differentiation by targeting myocardin (Tan et al., 2018).

In summary, our findings demonstrated that a high-salt diet fed to rats induced transcript expression changes in BASMCs. These differentially expressed transcripts were enriched in pathways involved in cellular morphological and construal plasticity, autophagy, and endocrine regulation. These results provide a deeper understanding of the development of high-salt-induced hypertension at the molecular and protein pathway levels and suggest areas that may be targeted for the discovery of new drugs in the treatment of hypertension.

## Conclusions

These genetic transcript changes in the BASMCs derived from high-salt intake–induced hypertensive rats may provide critical information about multiple cellular processes and biological functions that occur during the development of cerebrovascular disorders and provide potential new targets to help control or block the development of hypertension.

##  Supplemental Information

10.7717/peerj.9849/supp-1 Figure S1 Principal components analysis (PCA) visualization Heterogeneity of RNAseq data between control and high-salt diet groups is shown in the PCA figure.

10.7717/peerj.9849/supp-2 Table S1 Significantly upregulated and downregulated transcripts in high-salt diet group compared to control group Significance was statistically performed by comparing with regular diet control group. LogFC indicates the log of the fold change.

10.7717/peerj.9849/supp-3 Supplemental Information 3 Raw data of expression levels of 10 hub genes transcripts in qPCR verification experiment

## Additional Information and Declarations

### DNA Deposition

The following information was supplied regarding the deposition of DNA sequences: The raw data of RNA-seq are available at GEO: GSE147945.

### Data Availability

The following information was supplied regarding data availability: The overview over the up- and down-regulated transcripts are available as Table S1, and the raw data of qPCR are available in the Supplemental Files.

## References

[ref-1] Aher A, Kok M, Sharma A, Rai A, Olieric N, Kapitein LC, Steinmetz MO, Dogterom M, Akhmanova A (2018). CLASP suppresses microtubule catastrophes through a single TOG domain. Developmental Cell.

[ref-2] Arribas SM, Costa R, Salomone S, Morel N, Godfraind T, McGrath JC (1999). Functional reduction and associated cellular rearrangement in SHRSP rat basilar arteries are affected by salt load and calcium antagonist treatment. Journal of Cerebral Blood Flow and Metabolism.

[ref-3] Barnes JN (2017). Sex-specific factors regulating pressure and flow. Experimental Physiology.

[ref-4] Basheer WA, Harris BS, Mentrup HL, Abreha M, Thames EL, Lea JB, Swing DA, Copeland NG, Jenkins NA, Price RL, Matesic LE (2015). Cardiomyocyte-specific overexpression of the ubiquitin ligase Wwp1 contributes to reduction in Connexin 43 and arrhythmogenesis. Journal of Molecular and Cellular Cardiology.

[ref-5] Bkaily G, Simon Y, Menkovic I, Bkaily C, Jacques D (2018). High salt-induced hypertrophy of human basilar artery vascular smooth muscle cells associated with a decrease in glycocalyx. Journal of Molecular and Cellular Cardiology.

[ref-6] Boengler K, Schulz R (2017). Connexin 43 and Mitochondria in Cardiovascular Health and Disease. Advances in Experimental Medicine and Biology.

[ref-7] Bol M, Wang N, De Bock M, Wacquier B, Decrock E, Vadim Krysko D, Bultynck G, Dupont G, Vande Voorde J, Leybaert L (2017). At the cross-point of connexins, calcium, and ATP: blocking hemichannels inhibits vasoconstriction of rat small mesenteric arteries. Cardiovascular Research.

[ref-8] Bouguenina H, Salaun D, Mangon A, Muller L, Baudelet E, Camoin L, Tachibana T, Cianférani S, Audebert S, Verdier-Pinard P, Badache A (2017). EB1-binding-myomegalin protein complex promotes centrosomal microtubules functions. Proceedings of the National Academy of Sciences of the United States of America.

[ref-9] Bray NL, Pimentel H, Melsted P, Pachter L (2016). Near-optimal probabilistic RNA-seq quantification. Nature Biotechnology.

[ref-10] Chin CH, Chen SH, Wu HH, Ho CW, Ko MT, Lin CY (2014). cytoHubba: identifying hub objects and sub-networks from complex interactome. BMC Systems Biology.

[ref-11] Crestani S, Webb RC, Da Silva-Santos JE (2017). High-salt intake augments the activity of the RhoA/ROCK pathway and reduces intracellular calcium in arteries from rats. American Journal of Hypertension.

[ref-12] Davis H, Bardsley EN, Paterson DJ (2018). Transcriptional profiling of stellate ganglia from normotensive and spontaneously hypertensive rat strains. Scientific Data.

[ref-13] Egan Benova T, Szeiffova Bacova B, Viczenczova C, Diez E, Barancik M, Tribulova N (2016). Protection of cardiac cell-to-cell coupling attenuate myocardial remodeling and proarrhythmia induced by hypertension. Physiological Research.

[ref-14] Ginnan R, Zou X, Pfleiderer PJ, Mercure MZ, Barroso M, Singer HA (2013). Vascular smooth muscle cell motility is mediated by a physical and functional interaction of Ca^2+^/calmodulin-dependent protein kinase II *δ*2 and Fyn. Journal of Biological Chemistry.

[ref-15] Goetsch SC, Hawke TJ, Gallardo TD, Richardson JA, Garry DJ (2003). Transcriptional profiling and regulation of the extracellular matrix during muscle regeneration. Physiological Genomics.

[ref-16] Grillo A, Salvi L, Coruzzi P, Salvi P, Parati G (2019). Sodium Intake and Hypertension. Nutrients.

[ref-17] Iadecola C, Davisson RL (2008). Hypertension and cerebrovascular dysfunction. Cell Metabolism.

[ref-18] Ikezu T, Chen C, DeLeo AM, Zeldich E, Fallin MD, Kanaan NM, Lunetta KL, Abraham CR, Logue MW, Farrer LA (2018). Tau phosphorylation is impacted by rare AKAP9 mutations associated with Alzheimer disease in African Americans. Journal of Neuroimmune Pharmacology.

[ref-19] Jiang W, Ye L, Yang Y, Wang P, Pan W, Du J, Shen B, Wang K (2019). TRPP2 associates with STIM1 to regulate cerebral vasoconstriction and enhance high-salt intake–induced hypertensive cerebrovascular spasm. Hypertension Research.

[ref-20] Josipovic I, Fork C, Preussner J, Prior KK, Iloska D, Vasconez AE, Labocha S, Angioni C, Thomas D, Ferreirós N, Looso M, Pullamsetti SS, Geisslinger G, Steinhilber D, Brandes RP, Leisegang MS (2016). PAFAH1B1 and the lncRNA NONHSAT073641 maintain an angiogenic phenotype in human endothelial cells. Acta Physiologica.

[ref-21] Karppanen H, Mervaala E (2006). Sodium intake and hypertension. Progress in Cardiovascular Diseases.

[ref-22] Kawabe J, Okumura S, Lee MC, Sadoshima J, Ishikawa Y (2004). Translocation of caveolin regulates stretch-induced ERK activity in vascular smooth muscle cells. The American Journal of Physiology-Heart and Circulatory Physiology.

[ref-23] Kunin M, Dmitrieva NI, Gallazzini M, Shen RF, Wang G, Burg MB, Ferraris JD (2010). Mediator of DNA damage checkpoint 1 (MDC1) contributes to high NaCl-induced activation of the osmoprotective transcription factor TonEBP/OREBP. PLOS ONE.

[ref-24] Lacolley P, Regnault V, Nicoletti A, Li Z, Michel JB (2012). The vascular smooth muscle cell in arterial pathology: a cell that can take on multiple roles. Cardiovascular Research.

[ref-25] Liu M, Wu B, Wang WZ, Lee LM, Zhang SH, Kong LZ (2007). Stroke in China: epidemiology, prevention, and management strategies. Lancet Neurology.

[ref-26] Long L, Yang X, Southwood M, Lu J, Marciniak SJ, Dunmore BJ, Morrell NW (2013). Chloroquine prevents progression of experimental pulmonary hypertension via inhibition of autophagy and lysosomal bone morphogenetic protein type II receptor degradation. Circulation Research.

[ref-27] Nickenig G, Strehlow K, Roeling J, Zolk O, Knorr A, Böhm M (1998). Salt induces vascular AT1 receptor overexpression in vitro and in vivo. Hypertension.

[ref-28] Nielsen MS, Axelsen LN, Sorgen PL, Verma V, Delmar M, Holstein-Rathlou NH (2012). Gap junctions. Comprehensive Physiology.

[ref-29] Padmanabhan S, Joe B (2017). Towards precision medicine for hypertension: a review of genomic, epigenomic, and microbiomic effects on blood pressure in experimental rat models and humans. Physiological Reviews.

[ref-30] Pertea M, Kim D, Pertea GM, Leek JT, Salzberg SL (2016). Transcript-level Expression Analysis of RNA-seq Experiments With HISAT, StringTie and Ballgown. Nature Protocols.

[ref-31] Robinson MD, McCarthy DJ, Smyth GK (2010). edgeR: a Bioconductor package for differential expression analysis of digital gene expression data. Bioinformatics.

[ref-32] Russo A, Di Gaetano C, Cugliari G, Matullo G (2018). Advances in the genetics of hypertension: the effect of rare variants. International Journal of Molecular Sciences.

[ref-33] Sayas CL, Basu S, Van der Reijden M, Bustos-Morán E, Liz M, Galjart N (2019). Distinct functions for mammalian CLASP1 and -2 during neurite and axon elongation. Frontiers in Cellular Neuroscience.

[ref-34] Schiff M, Delahaye A, Andrieux J, Sanlaville D, Vincent-Delorme C, Aboura A, Benzacken B, Bouquillon S, Elmaleh-Berges M, Labalme A, Passemard S, Perrin L, Manouvrier-Hanu S, Edery P, Verloes A, Drunat S (2010). Further delineation of the 17p13.3 microdeletion involving YWHAE but distal to PAFAH1B1: four additional patients. European Journal of Medical Genetics.

[ref-35] Sehgel NL, Sun Z, Hong Z, Hunter WC, Hill MA, Vatner DE, Vatner SF, Meininger GA (2015). Augmented vascular smooth muscle cell stiffness and adhesion when hypertension is superimposed on aging. Hypertension.

[ref-36] Shao Y, Wang LJ, Zhong L, Hong ML, Chen HM, Murphy RW, Wu D-D, Zhang Y-P, Che J (2015). Transcriptomes reveal the genetic mechanisms underlying ionic regulatory adaptations to salt in the crab-eating frog. Scientific Reports.

[ref-37] Soon WW, Hariharan M, Snyder MP (2013). High-throughput sequencing for biology and medicine. Molecular Systems Biology.

[ref-38] Starke R.M., Chalouhi N., Ding D., Raper D.M., Mckisic M.S., Owens G.K., Hasan D.M., Medel R., Dumont A.S. (2014). Vascular smooth muscle cells in cerebral aneurysm pathogenesis. Translational Stroke Research.

[ref-39] Streeter E, Hart J, Badoer E (2012). An investigation of the mechanisms of hydrogen sulfide-induced vasorelaxation in rat middle cerebral arteries. Naunyn-Schmiedebergs Archives of Pharmacology.

[ref-40] Su G, Morris JH, Demchak B, Bader GD (2014). Biological network exploration with Cytoscape 3. Current Protocols in Bioinformatics.

[ref-41] Szklarczyk D, Gable AL, Lyon D, Junge A, Wyder S, Huerta-Cepas J, Simonovic M, Doncheva NT, Morris JH, Bork P, Jensen LJ, Mering CV (2019). STRING v11: protein-protein association networks with increased coverage, supporting functional discovery in genome-wide experimental datasets. Nucleic Acids Research.

[ref-42] Tan Z, Li J, Zhang X, Yang X, Zhang Z, Yin K-J, Huang H (2018). P53 promotes retinoid acid-induced smooth muscle cell differentiation by targeting myocardin. Stem Cells and Development.

[ref-43] Taylor LE, Sullivan JC (2016). Sex differences in obesity-induced hypertension and vascular dysfunction: a protective role for estrogen in adipose tissue inflammation?. The American Journal of Physiology-Regulatory, Integrative and Comparative Physiology.

[ref-44] Touyz RM, Alves-Lopes R, Rios FJ, Camargo LL, Anagnostopoulou A, Arner A, Montezano AC (2018). Vascular smooth muscle contraction in hypertension. Cardiovascular Research.

[ref-45] Wang LJ, Liu WD, Zhang L, Ma KT, Zhao L, Shi W-Y, Zhang W-W, Wang Y-Z, Li L, Si JQ (2016). Enhanced expression of Cx43 and gap junction communication in vascular smooth muscle cells of spontaneously hypertensive rats. Molecular Medicine Reports.

[ref-46] Wolinsky H, Goldfischer S, Schiller B, Kasak LE (1973). Lysosomes in aortic smooth muscle cells. Effects of hypertension. American Journal of Pathology.

[ref-47] Xu H, Qing T, Shen Y, Huang J, Liu Y, Li J, Zhen T, Xing K, Zhu S, Luoa M, Luo M (2018). RNA-seq analyses the effect of high-salt diet in hypertension. Gene.

[ref-48] Zhao R, Zhou M, Li J, Wang X, Su K, Hu J, Ye Y, Zhu J, Zhang G, Wang K, Du J, Wang L, Shen B (2015). Increased TRPP2 expression in vascular smooth muscle cells from high-salt intake hypertensive rats: the crucial role in vascular dysfunction. Molecular Nutrition & Food Research.

[ref-49] Zhou M, Wang H, Zeng X, Yin P, Zhu J, Chen W, Li X, Wang L, Wang L, Liu Y, Liu J, Zhang M, Qi J, Yu S, Afshin A, Gakidou E, Glenn S, Sarah Krish V, Miller-Petrie MK, Mountjoy-Venning WC, Mullany EC, Boston Redford S, Liu H, Naghavi M, Hay SI, Wang L, Murray CJL, Liang X (2019). Mortality, morbidity, and risk factors in China and its provinces, 1990-2017: a systematic analysis for the Global Burden of Disease Study 2017. Lancet.

[ref-50] Zhu J, Yu M, Friesema J, Huang T, Roman RJ, Lombard JH (2005). Salt-induced ANG II suppression impairs the response of cerebral artery smooth muscle cells to prostacyclin. The American Journal of Physiology-Heart and Circulatory Physiology.

