# Supplementary figures and images for: Differentially expressed transcripts and associated protein pathways in basilar artery smooth muscle cells of the high-salt intake–induced hypertensive rat
Source: PeerJ. 2020 Oct 13;8:e9849. doi: 10.7717/peerj.9849 (PMC7566752; doi:10.7717/peerj.9849)

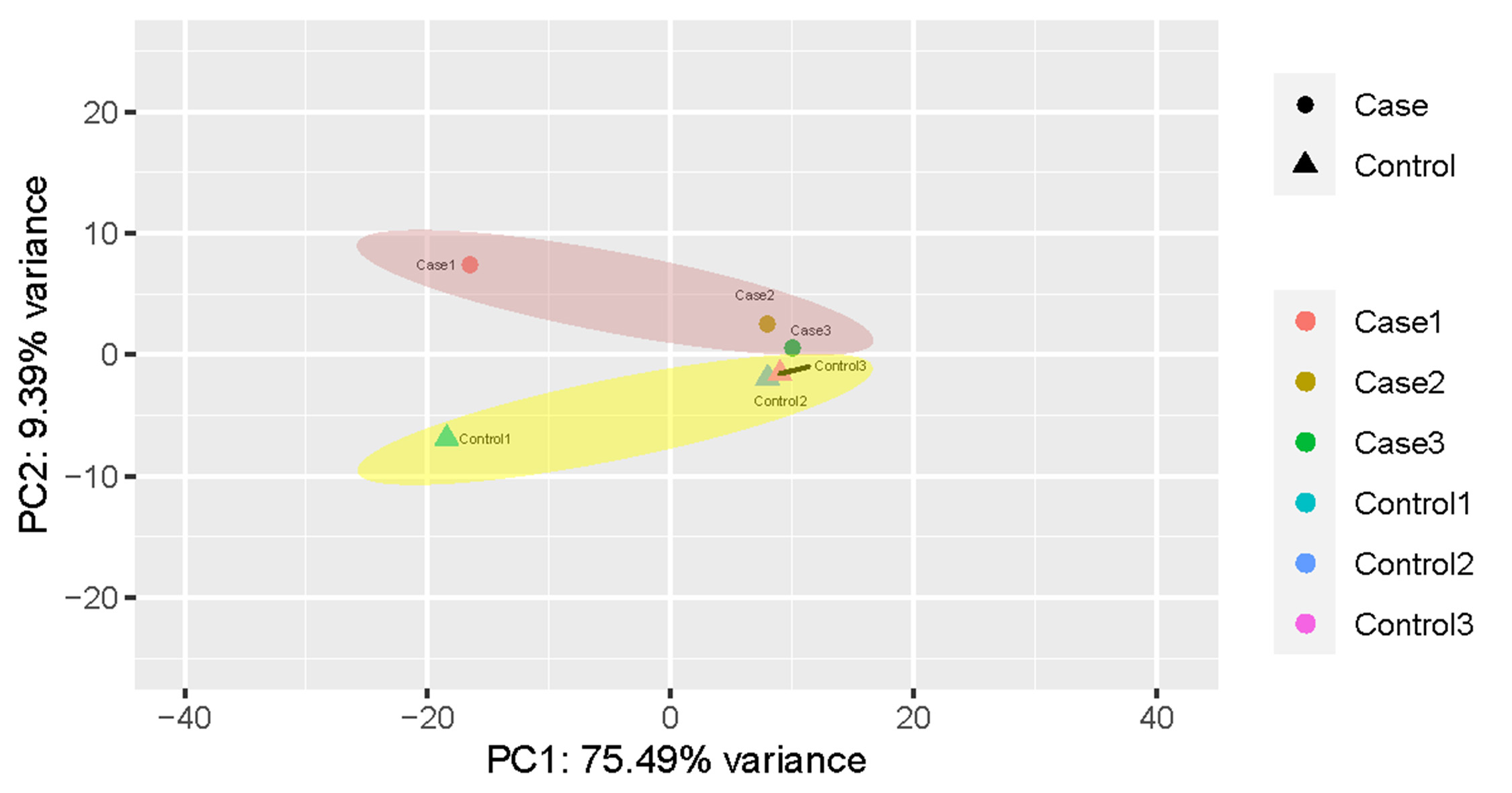

Supplement: Figure S1 — Heterogeneity of RNAseq data between control and high-salt diet groups is shown in the PCA figure. [file peerj-08-9849-s001.jpg]
