# Supplementary material for: Differentially expressed transcripts and associated protein pathways in basilar artery smooth muscle cells of the high-salt intake–induced hypertensive rat
Source: PeerJ. 2020 Oct 13;8:e9849. doi: 10.7717/peerj.9849 (PMC7566752; doi:10.7717/peerj.9849)
Supplement: Table S1 — Significance was statistically performed by comparing with regular diet control group. LogFC indicates the log of the fold change. [file peerj-08-9849-s002.docx]

**Table S1. Significantly upregulated and downregulated transcripts in high-salt diet group compared to control group.**

| **Name** | **Change** | **logFC** | ***P* Value** |
| --- | --- | --- | --- |
| *Calu* | Down-regulated | -10.22474368 | 0.000792217 |
| *Stk38* | Down-regulated | -10.09951144 | 0.004715627 |
| *Flot1* | Down-regulated | -10.05221449 | 0.000821012 |
| *Fbrsl1* | Down-regulated | -10.05046747 | 7.69E-05 |
| *Usp1* | Down-regulated | -10.05026342 | 7.38E-06 |
| *Arhgap17* | Down-regulated | -9.948273984 | 0.000781219 |
| *Zfp346* | Down-regulated | -9.833519321 | 0.00096334 |
| *Epb41* | Down-regulated | -9.777465616 | 0.007010031 |
| *Syk* | Down-regulated | -9.716150245 | 0.007620134 |
| *Rps18* | Down-regulated | -9.692045733 | 0.009372411 |
| *Sgk1* | Down-regulated | -9.413838567 | 0.00226084 |
| *LOC100911734* | Down-regulated | -9.404602689 | 0.014305977 |
| *Elmo1* | Down-regulated | -9.394676525 | 0.01096299 |
| *Rnf123* | Down-regulated | -9.241765182 | 0.000168656 |
| *LOC100911881* | Down-regulated | -9.226767856 | 0.01331213 |
| *NEWGENE_1562258* | Down-regulated | -9.063473945 | 0.003966426 |
| *St6gal1* | Down-regulated | -9.026200815 | 0.016613822 |
| *Hus1* | Down-regulated | -8.96260836 | 0.005920015 |
| *Adamts13* | Down-regulated | -8.871343832 | 0.004554143 |
| *Aqp4* | Down-regulated | -8.842544143 | 0.016595774 |
| *Clock* | Down-regulated | -8.662905803 | 0.031430434 |
| *4-Sep* | Down-regulated | -8.637665447 | 0.024854961 |
| *Wdr48* | Down-regulated | -8.617289013 | 0.000950125 |
| *Ldb3* | Down-regulated | -8.615663304 | 0.006058956 |
| *Cyhr1* | Down-regulated | -8.605392308 | 0.009659076 |
| *Tp53* | Down-regulated | -8.596552153 | 0.033844722 |
| *Col6a1* | Down-regulated | -8.587331084 | 0.034218453 |
| *Atat1* | Down-regulated | -8.50103059 | 0.001465303 |
| *Fam83h* | Down-regulated | -8.463816709 | 0.011822238 |
| *RT1-A1* | Down-regulated | -8.405667573 | 0.010320231 |
| *Fam110a* | Down-regulated | -8.396354343 | 0.03159461 |
| *AABR07009106.1* | Down-regulated | -8.344723317 | 0.011408706 |
| *Plekhg2* | Down-regulated | -8.332890849 | 0.01316648 |
| *Kctd15* | Down-regulated | -8.235725174 | 0.011973057 |
| *Wars* | Down-regulated | -8.209867435 | 0.049146039 |
| *Txnl1* | Down-regulated | -8.167960436 | 0.015840686 |
| *Cpne7* | Down-regulated | -8.155826313 | 0.039591112 |
| *Gpr155* | Down-regulated | -8.150182788 | 0.004017804 |
| *Hspa2* | Down-regulated | -8.144058258 | 0.015622751 |
| *Kif20b* | Down-regulated | -8.113324487 | 0.009401391 |
| *Eno1* | Down-regulated | -8.111264767 | 0.002752213 |
| *Scarb1* | Down-regulated | -8.0591185 | 0.020109342 |
| *Birc3* | Down-regulated | -8.009101993 | 0.017896858 |
| *Rbfa* | Down-regulated | -8.007096132 | 0.045190647 |
| *Sec24c* | Down-regulated | -8.002992344 | 0.008543069 |
| *Mrgbp* | Down-regulated | -7.977672609 | 0.014766654 |
| *Egf* | Down-regulated | -7.931111523 | 0.003872411 |
| *Parn* | Down-regulated | -7.913853328 | 0.019562463 |
| *Arap2* | Down-regulated | -7.906568282 | 0.020426877 |
| *Slc28a1* | Down-regulated | -7.86327979 | 0.003847625 |
| *Lmo7* | Down-regulated | -7.812147756 | 0.023303338 |
| *LOC100362027* | Down-regulated | -7.805698415 | 2.68E-06 |
| *Slfn2* | Down-regulated | -7.787049289 | 0.005339546 |
| *Gsap* | Down-regulated | -7.751156791 | 0.017984246 |
| *Hectd2* | Down-regulated | -7.726026576 | 0.024088949 |
| *Cep85l* | Down-regulated | -7.705466105 | 0.025030809 |
| *Msh5* | Down-regulated | -7.609950266 | 0.028162263 |
| *Ppp1ca* | Down-regulated | -7.589479973 | 0.039115833 |
| *Zfp787* | Down-regulated | -7.573614875 | 0.031781832 |
| *AABR07032520.1* | Down-regulated | -7.509658322 | 0.032106927 |
| *Cyp4f37* | Down-regulated | -7.488454941 | 0.009370369 |
| *RT1-M6-2* | Down-regulated | -7.475730613 | 0.009064276 |
| *Obscn* | Down-regulated | -7.456767088 | 0.040381976 |
| *Slc7a3* | Down-regulated | -7.446338126 | 0.025829496 |
| *Fubp3* | Down-regulated | -7.431885633 | 0.033247175 |
| *AC107446.3* | Down-regulated | -7.419468601 | 0.044483724 |
| *Arl15* | Down-regulated | -7.385350071 | 0.034791298 |
| *Rfc1* | Down-regulated | -7.317568281 | 0.036761355 |
| *LOC103694872* | Down-regulated | -7.311120139 | 0.013693084 |
| *AABR07029803.1* | Down-regulated | -7.271957503 | 0.043366664 |
| *Nrcam* | Down-regulated | -7.270647849 | 0.0381521 |
| *Cd101* | Down-regulated | -7.219482181 | 0.043699796 |
| *AC109901.2* | Down-regulated | -7.214145666 | 0.049933898 |
| *Cd300a* | Down-regulated | -7.175425272 | 0.049406783 |
| *Tmbim6* | Down-regulated | -7.17293094 | 0.043989499 |
| *Pold2* | Down-regulated | -7.169219432 | 0.042722039 |
| *Uimc1* | Down-regulated | -7.124073635 | 0.045554877 |
| *Slc17a7* | Down-regulated | -7.119441323 | 0.048262198 |
| *Tdrd7* | Down-regulated | -7.070827613 | 0.030301807 |
| *LOC689986* | Down-regulated | -7.05474734 | 0.036745729 |
| *Uck2* | Down-regulated | -7.017686074 | 0.03222442 |
| *Tgfbr3l* | Down-regulated | -7.006688136 | 0.021762498 |
| *Gad1* | Down-regulated | -7.005589771 | 0.049775401 |
| *Col6a6* | Down-regulated | -7.002779191 | 0.021627193 |
| *Zfp41* | Down-regulated | -6.992830068 | 0.009698012 |
| *Gabra3* | Down-regulated | -6.939571012 | 0.023093575 |
| *Arc* | Down-regulated | -6.92222868 | 0.016234562 |
| *Dnai1* | Down-regulated | -6.909907841 | 0.014614665 |
| *AABR07013922.1* | Down-regulated | -6.892470994 | 0.020093991 |
| *LOC500007* | Down-regulated | -6.889528747 | 0.017398252 |
| *LOC103694169* | Down-regulated | -6.884802243 | 0.015521471 |
| *Prkd3* | Down-regulated | -6.876271646 | 0.043285515 |
| *Atat1* | Down-regulated | -6.868460622 | 0.039056321 |
| *Kif21a* | Down-regulated | -6.846612466 | 0.024907439 |
| *Dcst1* | Down-regulated | -6.814108143 | 0.017864593 |
| *Pafah1b1* | Down-regulated | -6.811791473 | 0.044484847 |
| *Laptm5* | Down-regulated | -6.644406437 | 0.000732495 |
| *RT1-M6-1* | Down-regulated | -6.580355326 | 0.021770141 |
| *AABR07065139.1* | Down-regulated | -6.561826195 | 0.033232994 |
| *Lrrc24* | Down-regulated | -6.497560462 | 0.018516902 |
| *Scn2a* | Down-regulated | -6.477865827 | 0.02649925 |
| *Hist1h2bk* | Down-regulated | -6.44791419 | 0.03439013 |
| *Slc17a3* | Down-regulated | -6.342555449 | 0.031910856 |
| *Pnpla2* | Down-regulated | -6.326783882 | 0.000865823 |
| *Gpr55* | Down-regulated | -6.271637079 | 0.032108908 |
| *Tctex1d4* | Down-regulated | -6.239146443 | 0.041063406 |
| *RT1-DMa* | Down-regulated | -6.233742402 | 0.041559476 |
| *LOC689986* | Down-regulated | -6.131945235 | 0.046325915 |
| *AABR07062170.1* | Down-regulated | -6.104513454 | 0.042437677 |
| *Ggcx* | Down-regulated | -6.057361788 | 0.048816295 |
| *Rpl3* | Down-regulated | -6.032439487 | 0.045604341 |
| *Tmbim7* | Down-regulated | -5.957526008 | 0.043614341 |
| *Gtf2i* | Down-regulated | -5.940325806 | 0.047758152 |
| *Prss46* | Down-regulated | -5.929399056 | 0.047068762 |
| *Lamc1* | Down-regulated | -5.846277559 | 0.02681597 |
| *Abca7* | Down-regulated | -5.761767396 | 0.040499334 |
| *AABR07033287.1* | Down-regulated | -5.688252914 | 0.026618098 |
| *4-Sep* | Down-regulated | -5.64174321 | 0.034369879 |
| *Igf1* | Down-regulated | -5.613259989 | 0.008720045 |
| *Acsl6* | Down-regulated | -5.538174739 | 0.017068829 |
| *AABR07052664.1* | Down-regulated | -5.329299415 | 0.010810446 |
| *RT1-CE1* | Down-regulated | -5.288315453 | 0.02126259 |
| *RGD1565844* | Down-regulated | -5.280122917 | 0.024470195 |
| *Slc7a10* | Down-regulated | -5.237788767 | 0.028187884 |
| *Rpl39l* | Down-regulated | -5.204075951 | 0.036549294 |
| *Med12l* | Down-regulated | -5.044441678 | 0.033832012 |
| *Arl6ip1* | Down-regulated | -4.941298319 | 0.030552252 |
| *Il1rl2* | Down-regulated | -4.916596477 | 0.035488862 |
| *Cd200* | Down-regulated | -4.858781083 | 0.036398047 |
| *Cdkl3* | Down-regulated | -4.852064573 | 0.022368856 |
| *Kcnmb1* | Down-regulated | -4.7770013 | 0.048505508 |
| *LOC100363469* | Down-regulated | -4.761846981 | 0.01139666 |
| *Gabbr1* | Down-regulated | -4.741467127 | 0.038657831 |
| *Haus7* | Down-regulated | -4.695572656 | 0.043886923 |
| *Snx30* | Down-regulated | -4.665926463 | 0.047418663 |
| *Supv3l1* | Down-regulated | -4.620264175 | 0.024458791 |
| *Mmel1* | Down-regulated | -4.587864167 | 0.045752014 |
| *Tpm3* | Down-regulated | -4.4832932 | 0.032869442 |
| *Fkbp10* | Down-regulated | -4.291067992 | 0.030081101 |
| *Mia2* | Down-regulated | -4.251624958 | 0.035355722 |
| *Ebf1* | Down-regulated | -4.213230045 | 0.04709004 |
| *Rnpc3* | Down-regulated | -4.051197678 | 0.023451614 |
| *AABR07028446.1* | Down-regulated | -3.930927239 | 0.049836544 |
| *Wnk1* | Down-regulated | -3.902981627 | 0.022812053 |
| *Anks1a* | Down-regulated | -3.786994302 | 0.01004266 |
| *AABR07045307.1* | Down-regulated | -3.749785762 | 0.035571313 |
| *Casr* | Down-regulated | -3.730574433 | 0.042498132 |
| *LOC103692170* | Down-regulated | -3.629102874 | 0.032031144 |
| *Thsd7b* | Down-regulated | -3.580705962 | 0.035397787 |
| *Asgr2* | Down-regulated | -2.798969606 | 0.024709878 |
| *Ggnbp2* | Down-regulated | -2.493630531 | 0.035924905 |
| *Akap9* | Down-regulated | -2.339949944 | 0.023025188 |
| *Thbs4* | Down-regulated | -2.250736197 | 0.039318976 |
| *Macf1* | Down-regulated | -2.217291835 | 0.001772501 |
| *Cfh* | Down-regulated | -2.125490034 | 0.038310286 |
| *Rsrp1* | Down-regulated | -2.015500208 | 0.002651075 |
| *Il9r* | Down-regulated | -2.007836549 | 0.048969257 |
| *LOC100910163* | Down-regulated | -1.840034364 | 0.01550544 |
| *Kank1* | Down-regulated | -1.742021531 | 0.012875952 |
| *NEWGENE_1308171* | Down-regulated | -1.574201785 | 0.007270726 |
| *Robo2* | Down-regulated | -1.408619369 | 0.04483601 |
| *AC141102.1* | Down-regulated | -1.404587343 | 0.015866172 |
| *Aspnl1* | Down-regulated | -1.375583651 | 0.012430324 |
| *Ndufa2* | Down-regulated | -1.170015937 | 0.0401316 |
| *Rbm3* | Down-regulated | -1.122480338 | 0.047714399 |
| *Gja1* | Up-regulated | 1.039164861 | 0.041530831 |
| *Tmcc2* | Up-regulated | 1.115001642 | 0.041115268 |
| *Slc26a2* | Up-regulated | 1.251513246 | 0.045613877 |
| *Supt6h* | Up-regulated | 1.25835844 | 0.043076805 |
| *Shisa3* | Up-regulated | 1.274741104 | 0.039442178 |
| *Arhgap17* | Up-regulated | 1.382901908 | 0.027057356 |
| *Parvb* | Up-regulated | 1.619973384 | 0.041424633 |
| *Slc4a4* | Up-regulated | 1.704936118 | 0.020273358 |
| *Rapgef3* | Up-regulated | 1.826434514 | 0.044806216 |
| *Slit2* | Up-regulated | 1.871613339 | 0.040243252 |
| *Slc5a6* | Up-regulated | 1.961773593 | 0.024968721 |
| *Exoc3* | Up-regulated | 2.136543173 | 0.042492914 |
| *Rgl1* | Up-regulated | 2.270113994 | 0.034264523 |
| *Gpam* | Up-regulated | 2.319178997 | 0.049254037 |
| *Thyn1* | Up-regulated | 2.363590727 | 0.019086969 |
| *Fras1* | Up-regulated | 2.791307742 | 0.045264718 |
| *AABR07015057.1* | Up-regulated | 2.793619048 | 0.04493156 |
| *AABR07015081.2* | Up-regulated | 2.79361905 | 0.044936729 |
| *Acaca* | Up-regulated | 2.966746778 | 0.013546308 |
| *Tjp3* | Up-regulated | 2.968706543 | 0.045766444 |
| *LOC100359600* | Up-regulated | 3.048265638 | 0.034314665 |
| *Map3k7* | Up-regulated | 3.16740487 | 0.007449467 |
| *Rai14* | Up-regulated | 3.321735907 | 0.037136638 |
| *RGD1304622* | Up-regulated | 3.495559149 | 0.040708247 |
| *Axl* | Up-regulated | 3.511477677 | 0.022529219 |
| *Prdm2* | Up-regulated | 3.611891233 | 0.043101233 |
| *Tuft1* | Up-regulated | 3.72569031 | 0.041736499 |
| *Samd4b* | Up-regulated | 3.827336652 | 0.030591839 |
| *Phf12* | Up-regulated | 3.894717592 | 0.031513196 |
| *Adamts7* | Up-regulated | 4.006952714 | 0.036822433 |
| *Grn* | Up-regulated | 4.032453615 | 0.006543169 |
| *Slc12a7* | Up-regulated | 4.075676368 | 0.044897796 |
| *AABR07013583.1* | Up-regulated | 4.292594247 | 0.024863777 |
| *Dhx8* | Up-regulated | 4.357822861 | 0.039274029 |
| *#N/A* | Up-regulated | 4.62184677 | 0.021754709 |
| *Dux4* | Up-regulated | 4.643363256 | 0.030511497 |
| *Mia2* | Up-regulated | 4.694702166 | 0.046825505 |
| *Cbx2* | Up-regulated | 4.730525069 | 0.010742422 |
| *Prelp* | Up-regulated | 4.736320166 | 0.028435903 |
| *LOC108348044* | Up-regulated | 4.961558871 | 0.016297967 |
| *Slc28a1* | Up-regulated | 5.013531929 | 0.04850328 |
| *Rrn3* | Up-regulated | 5.02508521 | 0.02489694 |
| *Rrn3* | Up-regulated | 5.025097899 | 0.024874587 |
| *AC114233.2* | Up-regulated | 5.096472848 | 0.048433372 |
| *Lhx3* | Up-regulated | 5.308558909 | 0.046980464 |
| *Zdhhc1* | Up-regulated | 5.331597938 | 0.030773677 |
| *Nipsnap2* | Up-regulated | 5.466289398 | 0.003866815 |
| *Galnt2* | Up-regulated | 5.486362308 | 0.011460822 |
| *Slc39a7* | Up-regulated | 5.509374433 | 0.028148448 |
| *Kcnq1* | Up-regulated | 5.599381788 | 0.03618559 |
| *Slc22a5* | Up-regulated | 5.682620144 | 0.025635107 |
| *LOC102552659* | Up-regulated | 5.711437119 | 0.004700852 |
| *Adgrl2* | Up-regulated | 5.78081146 | 0.0077176 |
| *Cd5l* | Up-regulated | 5.807056677 | 0.030936121 |
| *AC242859.3* | Up-regulated | 6.074530529 | 0.021409224 |
| *AABR07072539.5* | Up-regulated | 6.176513814 | 0.001804112 |
| *Taf5l* | Up-regulated | 6.545984848 | 0.021869033 |
| *LOC100910528* | Up-regulated | 6.564022244 | 0.011706455 |
| *Tpsg1* | Up-regulated | 6.642689607 | 0.028353338 |
| *Prg2* | Up-regulated | 6.669645174 | 0.005150741 |
| *Camta1* | Up-regulated | 6.72625345 | 0.013331109 |
| *Mpo* | Up-regulated | 6.79520704 | 0.038012548 |
| *AABR07002775.1* | Up-regulated | 6.820870641 | 0.01604781 |
| *Pmch* | Up-regulated | 6.851736084 | 0.016241928 |
| *Xylt2* | Up-regulated | 6.8655602 | 0.006913493 |
| *Epha3* | Up-regulated | 7.008655367 | 0.001221123 |
| *Safb* | Up-regulated | 7.092850872 | 0.030648402 |
| *RGD1302996* | Up-regulated | 7.13846222 | 0.045967003 |
| *Sla* | Up-regulated | 7.217623779 | 0.035346141 |
| *Clasp1* | Up-regulated | 7.277857292 | 0.046302049 |
| *Spint1* | Up-regulated | 7.423612538 | 0.009092115 |
| *RGD1561796* | Up-regulated | 7.427975636 | 0.048356497 |
| *St7l* | Up-regulated | 7.428587843 | 0.035115629 |
| *LOC100910196* | Up-regulated | 7.430269948 | 0.011537958 |
| *LOC100359515* | Up-regulated | 7.444894194 | 0.042325687 |
| *Klhdc1* | Up-regulated | 7.515462438 | 0.029099473 |
| *Galnt9* | Up-regulated | 7.528469153 | 0.011945057 |
| *Fam161a* | Up-regulated | 7.536836735 | 0.011182133 |
| *Rspo2* | Up-regulated | 7.538834184 | 0.021069106 |
| *AABR07048303.1* | Up-regulated | 7.591438238 | 0.00774994 |
| *LOC100911776* | Up-regulated | 7.597508464 | 0.040421491 |
| *Nav1* | Up-regulated | 7.601431416 | 0.033863426 |
| *Ly6h* | Up-regulated | 7.609407238 | 0.027428642 |
| *AABR07036374.1* | Up-regulated | 7.611781433 | 0.02064104 |
| *Stx12* | Up-regulated | 7.614736621 | 0.040520257 |
| *Mmp9* | Up-regulated | 7.617054295 | 0.034324849 |
| *Ireb2* | Up-regulated | 7.678005469 | 0.020036122 |
| *Slc40a1* | Up-regulated | 7.703796072 | 0.028357692 |
| *Cyp4f17* | Up-regulated | 7.725866985 | 0.010460414 |
| *Armh1* | Up-regulated | 7.729688727 | 0.034284682 |
| *AABR07064061.1* | Up-regulated | 7.81463345 | 0.00077395 |
| *Fyn* | Up-regulated | 7.822006234 | 0.028484544 |
| *Gne* | Up-regulated | 7.827135587 | 0.036373002 |
| *Abhd14a* | Up-regulated | 7.83374937 | 0.034996511 |
| *Pde4dip* | Up-regulated | 7.840917061 | 0.026603468 |
| *Dync1li2* | Up-regulated | 7.891445325 | 0.047900203 |
| *AABR07022022.1* | Up-regulated | 7.919808363 | 0.021697795 |
| *Ciita* | Up-regulated | 7.925410392 | 0.013825933 |
| *Abcc3* | Up-regulated | 7.932134406 | 0.018387751 |
| *AABR07053518.1* | Up-regulated | 7.9832411 | 0.009046045 |
| *Tmcc1* | Up-regulated | 7.993117423 | 0.005402503 |
| *Plcl1* | Up-regulated | 7.997473993 | 0.019915238 |
| *Tubgcp3* | Up-regulated | 7.998983675 | 0.017605851 |
| *4-Sep* | Up-regulated | 8.043134163 | 0.028169146 |
| *Tet1* | Up-regulated | 8.056168376 | 0.016476895 |
| *Maged1* | Up-regulated | 8.099529863 | 0.029055011 |
| *Ppp3cc* | Up-regulated | 8.153964692 | 0.003716183 |
| *NEWGENE_621351* | Up-regulated | 8.169187744 | 0.049289764 |
| *Ankrd6* | Up-regulated | 8.172938158 | 0.048049422 |
| *Btk* | Up-regulated | 8.210771773 | 0.046143455 |
| *AABR07044412.1* | Up-regulated | 8.216947884 | 0.046250856 |
| *AABR07015180.1* | Up-regulated | 8.246440597 | 0.012597596 |
| *LOC100911951* | Up-regulated | 8.265414255 | 0.044722936 |
| *Elf1* | Up-regulated | 8.286341074 | 0.043266465 |
| *Cd247* | Up-regulated | 8.293016816 | 0.043549138 |
| *LOC100909725* | Up-regulated | 8.296573302 | 0.003375864 |
| *Adcy9* | Up-regulated | 8.316237863 | 0.042159392 |
| *Grk3* | Up-regulated | 8.321310729 | 0.042185031 |
| *Hps1* | Up-regulated | 8.348033081 | 0.04118571 |
| *Fancb* | Up-regulated | 8.3607657 | 0.011035758 |
| *Mdc1* | Up-regulated | 8.381516052 | 0.010521468 |
| *NEWGENE_1589866* | Up-regulated | 8.389886604 | 0.012382762 |
| *Dclk2* | Up-regulated | 8.401322838 | 0.039093049 |
| *Zmym6* | Up-regulated | 8.414082395 | 0.012953637 |
| *Anpep* | Up-regulated | 8.416740603 | 0.010071682 |
| *Gpm6a* | Up-regulated | 8.432374602 | 0.037884741 |
| *Sptssb* | Up-regulated | 8.4927066 | 0.012031076 |
| *Il4i1* | Up-regulated | 8.500991912 | 0.008907891 |
| *Begain* | Up-regulated | 8.508741129 | 0.018732466 |
| *Lig3* | Up-regulated | 8.534061296 | 0.009437278 |
| *Dopey2* | Up-regulated | 8.538579313 | 0.033856459 |
| *LOC103693189* | Up-regulated | 8.554022189 | 0.000984373 |
| *AABR07006627.1* | Up-regulated | 8.556710671 | 0.009526767 |
| *Hdac3* | Up-regulated | 8.581963572 | 0.032338394 |
| *Shank3* | Up-regulated | 8.620182324 | 0.031403674 |
| *Zfp438* | Up-regulated | 8.624316025 | 0.008891669 |
| *Alox12* | Up-regulated | 8.632217036 | 0.022676598 |
| *LOC103694867* | Up-regulated | 8.637023644 | 0.030967337 |
| *#N/A* | Up-regulated | 8.757635246 | 0.027740561 |
| *Nedd4l* | Up-regulated | 8.759018001 | 0.008917716 |
| *Ptpn12* | Up-regulated | 8.76782403 | 0.013062217 |
| *Cand2* | Up-regulated | 8.776333278 | 0.016541537 |
| *Ccz1b* | Up-regulated | 8.857047809 | 0.009068304 |
| *Stk38* | Up-regulated | 8.860120287 | 0.007558667 |
| *Slc38a5* | Up-regulated | 8.884189398 | 0.023853754 |
| *AABR07061134.1* | Up-regulated | 8.913425638 | 0.005126097 |
| *Baiap2l1* | Up-regulated | 8.958466803 | 0.00468067 |
| *Asgr2* | Up-regulated | 8.958857242 | 0.022272039 |
| *Sema3e* | Up-regulated | 9.000551112 | 0.02136831 |
| *LOC103689961* | Up-regulated | 9.01671569 | 0.004036191 |
| *Selenow* | Up-regulated | 9.016715704 | 0.004035264 |
| *Etnk2* | Up-regulated | 9.055920994 | 0.004410189 |
| *Wbp11* | Up-regulated | 9.068232019 | 0.020235115 |
| *Ranbp2* | Up-regulated | 9.076425972 | 0.019504793 |
| *Prkcb* | Up-regulated | 9.076610773 | 0.019680657 |
| *Ache* | Up-regulated | 9.085975297 | 0.000585987 |
| *Wdr44* | Up-regulated | 9.097208669 | 0.004044675 |
| *Nup155* | Up-regulated | 9.105601671 | 0.019134333 |
| *Odr4* | Up-regulated | 9.107756659 | 0.019108405 |
| *Cep63* | Up-regulated | 9.114315353 | 0.010170037 |
| *Rhobtb1* | Up-regulated | 9.16301443 | 0.006596485 |
| *Mycbpap* | Up-regulated | 9.180192061 | 0.01777542 |
| *LOC108348078* | Up-regulated | 9.182313012 | 0.007090364 |
| *Naa35* | Up-regulated | 9.185332534 | 0.017767674 |
| *Erbb2* | Up-regulated | 9.186158531 | 0.017445259 |
| *Flot2* | Up-regulated | 9.279144998 | 0.016052211 |
| *Impad1* | Up-regulated | 9.372421391 | 0.00037362 |
| *Tnfrsf26* | Up-regulated | 9.496279019 | 0.012413329 |
| *Gns* | Up-regulated | 9.536250651 | 0.003377638 |
| *Ubac2* | Up-regulated | 9.560862757 | 0.0014607 |
| *Lamc1* | Up-regulated | 9.562727925 | 0.003941073 |
| *Pld1* | Up-regulated | 9.563681759 | 0.011655757 |
| *Ppp1r18* | Up-regulated | 9.597277765 | 0.01116223 |
| *Slc4a10* | Up-regulated | 9.60413272 | 0.000129797 |
| *Ralgapa1* | Up-regulated | 9.608300516 | 0.004874931 |
| *Med14* | Up-regulated | 9.695650891 | 0.00176717 |
| *Polr2m* | Up-regulated | 9.706037741 | 0.009790763 |
| *Nol6* | Up-regulated | 9.740695453 | 0.009377194 |
| *M6pr* | Up-regulated | 9.753866878 | 0.001587592 |
| *Cttn* | Up-regulated | 9.755389487 | 0.001151329 |
| *Zfp189* | Up-regulated | 9.834282769 | 0.000125469 |
| *Rock1* | Up-regulated | 9.880513771 | 0.008070255 |
| *Tmem255b* | Up-regulated | 9.892412372 | 0.001224518 |
| *Tcf12* | Up-regulated | 9.934344189 | 0.000164939 |
| *Asph* | Up-regulated | 9.990720811 | 5.19E-05 |
| *Spp1* | Up-regulated | 10.12668265 | 0.000856289 |
| *LOC100909481* | Up-regulated | 10.13130399 | 0.000588938 |
| *Camsap1* | Up-regulated | 10.18683104 | 0.000490261 |
| *Zfp260* | Up-regulated | 10.19192636 | 0.000578379 |
| *Myh9l1* | Up-regulated | 10.24498165 | 0.002722997 |
| *Gtf2ird1* | Up-regulated | 10.33415326 | 0.004398402 |
| *Mef2c* | Up-regulated | 10.39733022 | 0.00407658 |
| *6-Sep* | Up-regulated | 10.59326566 | 6.19E-05 |
| *Exoc2* | Up-regulated | 10.63894786 | 0.000158218 |
| *Ceacam1* | Up-regulated | 10.67477246 | 0.000571424 |
| *Ddx17* | Up-regulated | 10.71997926 | 0.000127256 |
| *Optn* | Up-regulated | 10.81807762 | 0.000108244 |
| *Omd* | Up-regulated | 10.84559499 | 0.002087367 |
| *Ralgds* | Up-regulated | 10.84964532 | 0.002166076 |
| *Thrb* | Up-regulated | 10.85206153 | 7.99E-05 |
| *LOC103692716* | Up-regulated | 11.04952985 | 0.001593979 |
| *LOC100912282* | Up-regulated | 11.08147239 | 4.19E-05 |
| *Actn4* | Up-regulated | 11.193741 | 0.001306664 |

Note: Significance was statistically performed by comparing with regular diet control group. LogFC indicates the log of the fold change.
